# Supplementary material for: Association between PAI-1 Polymorphisms and Ischemic Stroke in a South Korean Case-Control Cohort
Source: Int J Mol Sci. 2023 Apr 28;24(9):8041. doi: 10.3390/ijms24098041 (PMC10178745; doi:10.3390/ijms24098041)
Supplement: Supplementary file 1 [file ijms-24-08041-s001.zip › ijms-2322206-Supplementary Figure S1.pdf]

International Journal of
Molecular Sciences

MDPI

Linkage disequilibrium (LD) patterns of *PAI-1* polymorphisms

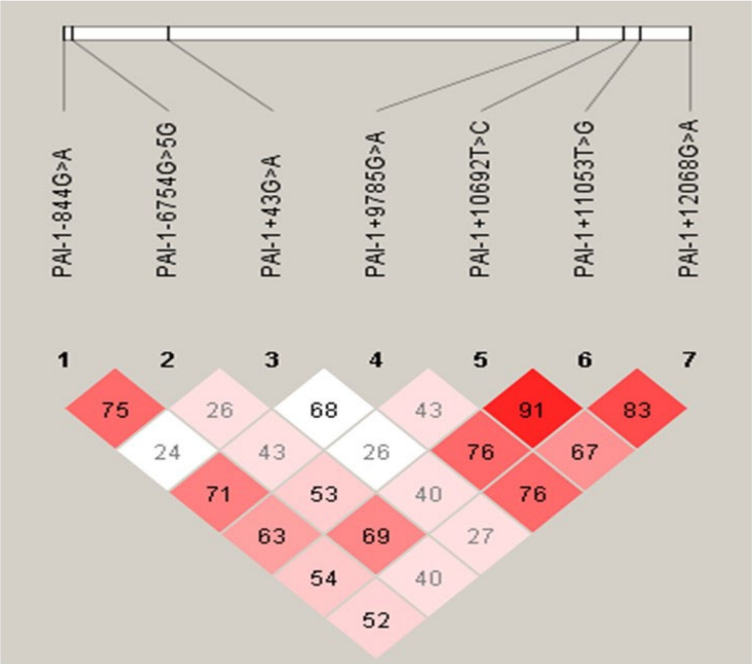

Supplementary Figure S1. The linkage disequilibrium (LD) block structure consisted of the seven SNPs located *PAI-1*. The LD pattern was derived from using both stroke patients and healthy control subjects. The LD block was defined by a D' value threshold of 0.8. The color scale ranges from red to white (color intensity decreases with decreasing D′ value). This locus was identified as one block, and the plot was generated by Haploview.
