# Supplementary material for: Association between PAI-1 Polymorphisms and Ischemic Stroke in a South Korean Case-Control Cohort
Source: Int J Mol Sci. 2023 Apr 28;24(9):8041. doi: 10.3390/ijms24098041 (PMC10178745; doi:10.3390/ijms24098041)
Supplement: Supplementary file 1 [file ijms-24-08041-s001.zip › ijms-2322206-Supplementary Table.pdf]

Supplementary Table S1. Haplotype for the *PAI-1* seven polymorphisms in ischemic stroke patients, metabolic syndrome patients and controls by MDR

Characteristics	MetS controls (2n=1528)	MetS patients (2n=470)	OR (95% CI)	*P*[a]	Stroke controls (2n=850)	Stroke patients (2n=1148)	OR (95% CI)	*P*[a]
***PAI-1* -844G>A/-675 4G>5G/43G>A/9785G>A/10692T>C/11053T>G/12068G>A**								
G-4G-G-G-T-T-G	24 (1.6)	10 (2.2)	1.000 (reference)		18 (2.1)	16 (1.4)	1.000 (reference)	
G-4G-G-G-T-T-A	15 (1.0)	7 (1.5)	1.120 (0.351 - 3.579)	1.000	8 (0.9)	17 (1.5)	2.391 (0.814 - 7.020)	0.122
G-4G-G-G-T-G-G	169 (11.0)	63 (13.4)	0.895 (0.405 - 1.976)	0.837	78 (9.1)	156 (13.6)	2.250 (1.088 - 4.652)	0.035
G-4G-G-G-T-G-A	11 (0.7)	0 (0.0)	0.101 (0.005 - 1.887)	0.089	3 (0.3)	8 (0.7)	3.000 (0.677 - 13.290)	0.177
G-4G-G-G-C-T-G	12 (0.8)	2 (0.3)	0.400 (0.075 - 2.123)	0.465	6 (0.8)	7 (0.6)	1.313 (0.364 - 4.730)	0.752
G-4G-G-G-C-T-A	120 (7.8)	31 (6.7)	0.620 (0.269 - 1.432)	0.261	70 (8.3)	81 (7.1)	1.302 (0.618 - 2.744)	0.570
G-4G-A-G-T-T-G	6 (0.4)	0 (0.0)	0.180 (0.009 - 3.487)	0.307	8 (1.0)	0 (0.0)	0.066 (0.004 - 1.234)	0.016
G-4G-A-G-T-G-G	5 (0.3)	2 (0.4)	0.960 (0.159 - 5.799)	1.000	7 (0.8)	0 (0.0)	0.075 (0.004 - 1.413)	0.031
G-4G-A-G-C-T-A	11 (0.7)	1 (0.2)	0.218 (0.025 - 1.923)	0.242	7 (0.8)	4 (0.3)	0.643 (0.158 - 2.610)	0.730
G-5G-G-G-T-T-G	25 (1.6)	7 (1.5)	0.672 (0.220 - 2.053)	0.578	21 (2.5)	13 (1.1)	0.696 (0.265 - 1.829)	0.624
G-5G-G-G-T-T-A	33 (2.1)	19 (4.1)	1.382 (0.546 - 3.499)	0.641	18 (2.1)	30 (2.6)	1.875 (0.769 - 4.574)	0.183
G-5G-G-G-T-G-G	60 (3.9)	11 (2.2)	0.440 (0.165 - 1.171)	0.120	36 (4.2)	35 (3.0)	1.094 (0.482 - 2.480)	0.838
G-5G-G-G-C-T-G	55 (3.6)	18 (3.7)	0.786 (0.316 - 1.951)	0.641	34 (4.0)	39 (3.4)	1.290 (0.571 - 2.917)	0.678
G-5G-G-G-C-T-A	244 (16.0)	64 (13.5)	0.630 (0.286 - 1.384)	0.273	131 (15.4)	168 (14.6)	1.443 (0.708 - 2.938)	0.364
G-5G-G-G-C-G-A	7 (0.5)	1 (0.2)	0.343 (0.037 - 3.163)	0.657	2 (0.2)	9 (0.8)	5.063 (0.949 - 27.000)	0.079
G-5G-G-A-C-T-G	32 (2.1)	8 (1.6)	0.600 (0.206 - 1.749)	0.420	12 (1.5)	29 (2.5)	2.719 (1.049 - 7.045)	0.058
G-5G-A-G-T-T-G	2 (0.2)	4 (0.8)	4.800 (0.754 - 30.560)	0.159	0 (0.0)	4 (0.4)	10.090 (0.504 - 202.100)	0.107
G-5G-A-G-T-T-A	4 (0.3)	0 (0.0)	0.259 (0.013 - 5.263)	0.556	2 (0.3)	5 (0.4)	2.813 (0.478 - 16.560)	0.410
G-5G-A-G-C-T-G	5 (0.3)	2 (0.5)	0.960 (0.159 - 5.799)	1.000	4 (0.5)	3 (0.3)	0.844 (0.163 - 4.358)	1.000
G-5G-A-G-C-T-A	51 (3.4)	17 (3.7)	0.800 (0.319 - 2.007)	0.641	24 (2.8)	48 (4.2)	2.250 (0.978 - 5.176)	0.060
A-4G-G-G-T-T-G	29 (1.9)	13 (2.8)	1.076 (0.401 - 2.885)	1.000	27 (3.2)	16 (1.4)	0.667 (0.267 - 1.664)	0.486
A-4G-G-G-T-T-A	15 (1.0)	1 (0.3)	0.160 (0.019 - 1.380)	0.080	4 (0.5)	12 (1.0)	3.375 (0.904 - 12.600)	0.076
A-4G-G-G-T-G-G	411 (26.9)	126 (26.8)	0.736 (0.343 - 1.580)	0.412	245 (28.8)	289 (25.2)	1.327 (0.662 - 2.658)	0.480
A-4G-G-G-T-G-A	10 (0.7)	9 (2.0)	2.160 (0.674 - 6.921)	0.239	9 (1.1)	13 (1.1)	1.625 (0.549 - 4.807)	0.423
A-4G-G-G-C-T-G	4 (0.3)	3 (0.6)	1.800 (0.339 - 9.555)	0.659	3 (0.3)	5 (0.4)	1.875 (0.385 - 9.124)	0.697
A-4G-G-G-C-T-A	38 (2.5)	10 (2.2)	0.632 (0.229 - 1.743)	0.438	17 (2.1)	35 (3.0)	2.316 (0.953 - 5.632)	0.075
A-4G-G-G-C-G-G	4 (0.2)	0 (0.0)	0.259 (0.013 - 5.263)	0.556	0 (0.0)	5 (0.4)	12.330 (0.632 - 240.600)	0.050
A-4G-A-G-T-G-G	26 (1.7)	2 (0.4)	**0.185 (0.037 - 0.930)**	**0.050**	22 (2.6)	3 (0.3)	**0.153 (0.039 - 0.611)**	**0.005**
A-5G-G-G-T-T-G	5 (0.4)	0 (0.0)	0.212 (0.011 - 4.196)	0.302	1 (0.2)	5 (0.4)	5.625 (0.592 - 53.410)	0.186
A-5G-G-G-C-T-A	27 (1.8)	11 (2.3)	0.978 (0.353 - 2.706)	1.000	0 (0.0)	37 (3.2)	**84.090 (4.774 - 1481.000)**	**<0.0001**
A-5G-A-G-C-T-A	3 (0.2)	3 (0.7)	2.400 (0.412 - 13.990)	0.370	0 (0.0)	7 (0.6)	**16.820 (0.890 - 317.900)**	**0.012**

MetS, metabolic syndrome; 95% CI, 95% confidence interval.
[a] Fisher's exact test.

Supplementary Table S2. **Haplotype for the *PAI-1* six polymorphisms in ischemic stroke patients, metabolic syndrome patients and controls by MDR**

Characteristics	MetS controls (2n=1528)	MetS patients (2n=470)	OR (95% CI)	*P*[a]	Controls (2n=850)	Stroke patients (2n=1148)	OR (95% CI)	*P*[a]
***PAI-1* -844G>A/-675 4G>5G/43G>A/10692T>C/11053T>G/12068G>A**								
G-4G-G-T-T-G	29 (1.9)	12 (2.5)	1.000 (reference)		21 (2.5)	19 (1.7)	1.000 (reference)	
G-4G-G-T-T-A	18 (1.2)	7 (1.4)	0.940 (0.312 - 2.830)	1.000	10 (1.2)	17 (1.5)	1.879 (0.693 - 5.097)	0.318
G-4G-G-T-G-G	170 (11.1)	65 (13.8)	0.924 (0.445 - 1.920)	0.851	77 (9.0)	158 (13.8)	**2.268 (1.151 - 4.467)**	**0.020**
G-4G-G-T-G-A	11 (0.7)	0 (0.0)	0.103 (0.006 - 1.881)	0.050	4 (0.4)	9 (0.8)	2.487 (0.657 - 9.418)	0.213
G-4G-G-C-T-G	17 (1.1)	2 (0.4)	0.284 (0.057 - 1.426)	0.189	8 (0.9)	9 (0.8)	1.243 (0.399 - 3.876)	0.777
G-4G-G-C-T-A	118 (7.8)	31 (6.6)	0.635 (0.291 - 1.386)	0.293	71 (8.3)	80 (7.0)	1.245 (0.620 - 2.503)	0.595
G-4G-A-T-T-G	7 (0.4)	0 (0.0)	0.157 (0.008 - 2.973)	0.169	8 (1.0)	0 (0.0)	0.065 (0.004 - 1.200)	0.015
G-4G-A-T-G-G	5 (0.4)	2 (0.4)	0.967 (0.164 - 5.692)	1.000	8 (1.0)	0 (0.0)	0.065 (0.004 - 1.200)	0.015
G-4G-A-C-T-G	1 (0.1)	2 (0.3)	4.833 (0.399 - 58.500)	0.234	3 (0.4)	0 (0.0)	0.158 (0.008 - 3.249)	0.243
G-4G-A-C-T-A	12 (0.8)	1 (0.2)	0.201 (0.023 - 1.726)	0.151	6 (0.7)	4 (0.4)	0.737 (0.180 - 3.017)	0.736
G-4G-A-C-G-G	3 (0.2)	0 (0.0)	0.337 (0.016 - 7.026)	0.551	1 (0.2)	2 (0.2)	2.211 (0.185 - 26.400)	0.607
G-5G-G-T-T-G	28 (1.8)	7 (1.5)	0.604 (0.208 - 1.757)	0.430	21 (2.5)	16 (1.4)	0.842 (0.343 - 2.070)	0.820
G-5G-G-T-T-A	32 (2.1)	20 (4.2)	1.510 (0.630 - 3.622)	0.387	17 (2.0)	30 (2.6)	1.950 (0.825 - 4.609)	0.137
G-5G-G-T-G-G	59 (3.9)	11 (2.3)	0.451 (0.178 - 1.143)	0.097	36 (4.2)	34 (3.0)	1.044 (0.480 - 2.272)	1.000
G-5G-G-T-G-A	9 (0.6)	1 (0.3)	0.269 (0.031 - 2.360)	0.419	5 (0.6)	5 (0.4)	1.105 (0.276 - 4.422)	1.000
G-5G-G-C-T-G	88 (5.8)	26 (5.5)	0.714 (0.320 - 1.593)	0.406	47 (5.5)	69 (6.0)	1.623 (0.787 - 3.344)	0.201
G-5G-G-C-T-A	247 (16.1)	62 (13.3)	0.607 (0.293 - 1.257)	0.220	134 (15.8)	167 (14.5)	1.377 (0.711 - 2.668)	0.399
G-5G-G-C-G-G	2 (0.1)	2 (0.4)	2.417 (0.304 - 19.200)	0.578	2 (0.2)	2 (0.2)	1.105 (0.141 - 8.640)	1.000
G-5G-G-C-G-A	8 (0.5)	1 (0.3)	0.302 (0.034 - 2.687)	0.414	3 (0.4)	9 (0.8)	3.316 (0.780 - 14.090)	0.113
G-5G-A-T-T-G	4 (0.2)	4 (0.8)	2.417 (0.518 - 11.280)	0.411	0 (0.0)	5 (0.4)	12.130 (0.629 - 234.000)	0.051
G-5G-A-T-T-A	4 (0.3)	0 (0.0)	0.262 (0.013 - 5.248)	0.561	4 (0.4)	4 (0.4)	1.105 (0.242 - 5.048)	1.000
G-5G-A-C-T-G	5 (0.3)	2 (0.4)	0.967 (0.164 - 5.692)	1.000	4 (0.4)	3 (0.2)	0.829 (0.164 - 4.192)	1.000
G-5G-A-C-T-A	51 (3.3)	17 (3.7)	0.806 (0.338 - 1.920)	0.659	23 (2.7)	48 (4.2)	**2.307 (1.041 - 5.109)**	**0.045**
A-4G-G-T-T-G	28 (1.9)	17 (3.6)	1.467 (0.595 - 3.621)	0.495	27 (3.2)	19 (1.7)	0.778 (0.331 - 1.828)	0.665
A-4G-G-T-T-A	16 (1.1)	1 (0.3)	0.151 (0.018 - 1.271)	0.083	5 (0.6)	12 (1.0)	2.653 (0.788 - 8.933)	0.149
A-4G-G-T-G-G	410 (26.8)	125 (26.5)	0.737 (0.365 - 1.487)	0.446	243 (28.6)	288 (25.1)	1.310 (0.688 - 2.494)	0.417
A-4G-G-T-G-A	10 (0.7)	11 (2.3)	2.658 (0.894 - 7.901)	0.098	10 (1.1)	13 (1.1)	1.437 (0.512 - 4.033)	0.603
A-4G-G-C-T-A	39 (2.5)	11 (2.3)	0.682 (0.264 - 1.761)	0.474	18 (2.1)	36 (3.2)	2.211 (0.954 - 5.120)	0.090
A-4G-G-C-G-G	4 (0.2)	0 (0.0)	0.262 (0.013 - 5.248)	0.561	0 (0.0)	5 (0.5)	12.130 (0.629 - 234.000)	0.051
A-4G-A-T-G-G	26 (1.7)	2 (0.4)	**0.186 (0.038 - 0.910)**	**0.033**	23 (2.7)	4 (0.3)	**0.192 (0.056 - 0.658)**	**0.008**
A-5G-G-T-T-G	5 (0.4)	0 (0.0)	0.215 (0.011 - 4.184)	0.306	1 (0.2)	4 (0.4)	4.421 (0.453 - 43.140)	0.346
A-5G-G-C-T-A	27 (1.8)	12 (2.6)	1.074 (0.413 - 2.796)	1.000	0 (0.0)	39 (3.4)	**87.100 (5.005 - 1516.000)**	**<0.0001**
A-5G-A-C-T-A	3 (0.2)	3 (0.7)	2.417 (0.426 - 13.720)	0.367	0 (0.0)	7 (0.6)	16.540 (0.885 - 309.200)	0.012

MetS, metabolic syndrome; 95% CI, 95% confidence interval.
[a] Fisher's exact test.

Supplementary Table S3. Haplotype for the *PAI-1* five and four polymorphisms in ischemic stroke patients, metabolic syndrome patients and controls by MDR

Characteristics	MetS controls (2n=1528)	MetS patients (2n=470)	OR (95% CI)	P[a]	Controls (2n=850)	Stroke patients (2n=1148)	OR (95% CI)	P[a]
***PAI-1* -844G>A/-675 4G>5G/10692T>C/11053T>G/12068G>A**								
G-4G-T-T-G	36 (2.4)	13 (2.7)	1.000 (reference)		29 (3.4)	20 (1.8)	1.000 (reference)	
G-4G-T-T-A	18 (1.2)	7 (1.5)	1.077 (0.366 - 3.169)	1.000	10 (1.1)	16 (1.4)	2.320 (0.876 - 6.146)	0.097
G-4G-T-G-G	174 (11.4)	66 (14.0)	1.050 (0.524 - 2.104)	1.000	87 (10.2)	155 (13.5)	**2.583 (1.379 - 4.838)**	**0.004**
G-4G-T-G-A	13 (0.8)	0 (0.0)	0.100 (0.006 - 1.805)	0.053	3 (0.4)	12 (1.0)	**5.800 (1.448 - 23.240)**	**0.016**
G-4G-C-T-G	17 (1.1)	3 (0.6)	0.489 (0.123 - 1.946)	0.364	11 (1.3)	8 (0.7)	1.055 (0.360 - 3.088)	1.000
G-4G-C-T-A	131 (8.6)	33 (7.0)	0.698 (0.333 - 1.463)	0.331	77 (9.0)	85 (7.4)	1.601 (0.837 - 3.060)	0.192
G-4G-C-G-G	5 (0.3)	3 (0.6)	1.662 (0.347 - 7.954)	0.674	2 (0.2)	5 (0.4)	3.625 (0.639 - 20.580)	0.223
G-5G-T-T-G	31 (2.0)	10 (2.2)	0.893 (0.344 - 2.320)	1.000	21 (2.4)	21 (1.8)	1.450 (0.632 - 3.329)	0.406
G-5G-T-T-A	36 (2.4)	21 (4.4)	1.615 (0.703 - 3.712)	0.300	22 (2.5)	34 (2.9)	2.241 (1.025 - 4.901)	0.051
G-5G-T-G-G	60 (3.9)	11 (2.4)	0.508 (0.206 - 1.253)	0.166	35 (4.2)	35 (3.0)	1.450 (0.693 - 3.032)	0.355
G-5G-T-G-A	8 (0.5)	1 (0.3)	0.346 (0.039 - 3.044)	0.431	5 (0.5)	5 (0.4)	1.450 (0.371 - 5.675)	0.729
G-5G-C-T-G	93 (6.1)	28 (5.9)	0.834 (0.389 - 1.787)	0.694	51 (6.0)	70 (6.1)	1.990 (1.014 - 3.907)	0.062
G-5G-C-T-A	298 (19.5)	79 (16.7)	0.734 (0.372 - 1.451)	0.361	158 (18.6)	217 (18.9)	**1.991 (1.087 - 3.649)**	**0.032**
G-5G-C-G-A	8 (0.5)	1 (0.2)	0.346 (0.039 - 3.044)	0.431	3 (0.4)	9 (0.8)	4.350 (1.045 - 18.100)	0.052
A-4G-T-T-G	31 (2.0)	16 (3.5)	1.429 (0.596 - 3.431)	0.507	27 (3.1)	20 (1.8)	1.074 (0.477 - 2.419)	1.000
A-4G-T-T-A	16 (1.0)	3 (0.6)	0.519 (0.130 - 2.078)	0.526	5 (0.6)	12 (1.1)	**3.480 (1.060 - 11.430)**	**0.049**
A-4G-T-G-G	436 (28.5)	127 (26.9)	0.807 (0.415 - 1.568)	0.594	265 (31.2)	295 (25.7)	1.614 (0.892 - 2.922)	0.136
A-4G-T-G-A	16 (1.0)	11 (2.3)	1.904 (0.703 - 5.155)	0.302	12 (1.4)	13 (1.1)	1.571 (0.596 - 4.143)	0.460
A-4G-C-T-G	8 (0.5)	2 (0.5)	0.692 (0.130 - 3.695)	1.000	5 (0.6)	6 (0.5)	1.740 (0.466 - 6.493)	0.507
A-4G-C-T-A	38 (2.5)	10 (2.2)	0.729 (0.284 - 1.870)	0.634	17 (2.1)	35 (3.1)	**2.985 (1.324 - 6.729)**	**0.010**
A-4G-C-G-G	4 (0.3)	0 (0.0)	0.300 (0.015 - 5.965)	0.561	0 (0.0)	5 (0.4)	**15.830 (0.828 - 302.500)**	**0.017**
A-5G-T-T-G	6 (0.4)	0 (0.0)	0.208 (0.011 - 3.950)	0.317	1 (0.2)	4 (0.4)	5.800 (0.602 - 55.840)	0.159
A-5G-T-G-G	7 (0.5)	4 (0.9)	1.582 (0.397 - 6.308)	0.712	1 (0.2)	12 (1.0)	**17.400 (2.092 - 144.800)**	**0.001**
A-5G-C-T-A	28 (1.9)	16 (3.5)	1.582 (0.654 - 3.827)	0.372	0 (0.0)	43 (3.8)	**125.200 (7.280 - 2153.000)**	**<0.0001**
***PAI-1* -844G>A/-675 4G>5G/43G>A/10692T>C**								
G-4G-G-T	222 (14.5)	84 (17.9)	1.000 (reference)		112 (13.2)	198 (17.2)	1.000 (reference)	
G-4G-G-C	143 (9.4)	36 (7.7)	0.665 (0.427 - 1.037)	0.081	82 (9.6)	94 (8.2)	**0.648 (0.445 - 0.945)**	**0.027**
G-4G-A-T	16 (1.1)	2 (0.5)	0.330 (0.074 - 1.468)	0.172	15 (1.7)	4 (0.3)	**0.151 (0.049 - 0.466)**	**0.0003**
G-4G-A-C	14 (0.9)	3 (0.6)	0.566 (0.159 - 2.021)	0.575	11 (1.3)	6 (0.5)	**0.309 (0.111 - 0.857)**	**0.022**
G-5G-G-T	131 (8.6)	37 (7.8)	0.747 (0.479 - 1.163)	0.226	77 (9.1)	86 (7.5)	**0.632 (0.430 - 0.929)**	**0.023**
G-5G-G-C	344 (22.5)	90 (19.2)	**0.691 (0.491 - 0.973)**	**0.035**	188 (22.1)	249 (21.7)	0.749 (0.555 - 1.011)	0.059
G-5G-A-T	11 (0.7)	8 (1.7)	1.922 (0.747 - 4.945)	0.192	6 (0.7)	12 (1.0)	1.131 (0.413 - 3.098)	1.000
G-5G-A-C	51 (3.3)	17 (3.6)	0.881 (0.482 - 1.611)	0.764	24 (2.9)	45 (3.9)	1.061 (0.614 - 1.833)	0.890
A-4G-G-T	461 (30.2)	152 (32.4)	0.871 (0.639 - 1.189)	0.423	281 (33.1)	331 (28.8)	**0.666 (0.503 - 0.883)**	**0.005**
A-4G-G-C	48 (3.2)	16 (3.4)	0.881 (0.474 - 1.636)	0.758	23 (2.7)	44 (3.9)	1.082 (0.621 - 1.885)	0.888
A-4G-A-T	36 (2.4)	1 (0.3)	**0.073 (0.010 - 0.544)**	**0.0004**	28 (3.3)	7 (0.6)	**0.141 (0.060 - 0.334)**	**<0.0001**
A-4G-A-C	4 (0.2)	0 (0.0)	0.293 (0.016 - 5.497)	0.578	0 (0.0)	3 (0.3)	3.967 (0.203 - 77.560)	0.555
A-5G-G-T	9 (0.6)	5 (1.0)	1.468 (0.478 - 4.509)	0.545	3 (0.3)	17 (1.5)	3.205 (0.919 - 11.180)	0.087
A-5G-G-C	33 (2.2)	14 (3.0)	1.121 (0.572 - 2.200)	0.729	0 (0.0)	40 (3.5)	**45.910 (2.794 - 754.300)**	**<0.0001**
A-5G-A-T	4 (0.3)	0 (0.0)	0.293 (0.016 - 5.497)	0.578	0 (0.0)	2 (0.1)	2.834 (0.135 - 59.590)	0.538
A-5G-A-C	0 (0.0)	5 (1.0)	**28.960 (1.583 - 529.900)**	**0.002**	0 (0.0)	9 (0.8)	**10.770 (0.621 - 186.900)**	**0.030**

MetS, metabolic syndrome; 95% CI, 95% confidence interval.
[a] Fisher's exact test.

Supplementary Table S4. Haplotype for the *PAI-1* three and two polymorphisms in ischemic stroke patients, metabolic syndrome patients and controls by MDR

**Characteristics**	**MetS controls (2n=1528)**	**MetS patients (2n=470)**	**OR (95% CI)**	***P*[a]**	**Controls (2n=850)**	**Stroke patients (2n=1148)**	**OR (95% CI)**	***P*[a]**
***PAI-1* -844G>A/-675 4G>5G/10692T>C**								
G-4G-T	238 (15.6)	86 (18.3)	1.000 (reference)		127 (15.0)	201 (17.6)	1.000 (reference)	
G-4G-C	157 (10.3)	39 (8.3)	0.688 (0.448 - 1.056)	0.091	92 (10.9)	101 (8.8)	0.694 (0.484 - 0.994)	0.054
G-5G-T	140 (9.2)	44 (9.4)	0.870 (0.572 - 1.323)	0.528	83 (9.8)	98 (8.6)	0.746 (0.517 - 1.077)	0.133
G-5G-C	396 (25.9)	107 (22.8)	0.748 (0.540 - 1.036)	0.092	212 (25.0)	294 (25.6)	0.876 (0.660 - 1.164)	0.387
A-4G-T	497 (32.6)	154 (32.8)	0.858 (0.632 - 1.164)	0.344	309 (36.4)	339 (29.5)	**0.693 (0.529 - 0.909)**	**0.008**
A-4G-C	52 (3.4)	16 (3.3)	0.852 (0.462 - 1.571)	0.652	23 (2.8)	47 (4.1)	1.291 (0.748 - 2.229)	0.416
A-5G-T	14 (0.9)	4 (0.9)	0.791 (0.253 - 2.469)	0.790	3 (0.3)	19 (1.6)	**4.002 (1.160 - 13.800)**	**0.021**
A-5G-C	32 (2.1)	19 (4.0)	1.643 (0.885 - 3.052)	0.131	0 (0.0)	49 (4.3)	**62.640 (3.827 - 1025.000)**	**<0.0001**
***PAI-1* -844G>A/-675 4G>5G**								
G-4G	398 (26.0)	125 (26.7)	1.000 (reference)		220 (25.9)	304 (26.5)	1.000 (reference)	
G-5G	534 (35.0)	152 (32.3)	0.906 (0.692 - 1.187)	0.490	295 (34.7)	390 (34.0)	0.957 (0.760 - 1.204)	0.725
A-4G	547 (35.8)	170 (36.1)	0.955 (0.732 - 1.246)	0.734	332 (39.1)	384 (33.5)	0.837 (0.667 - 1.051)	0.133
A-5G	49 (3.2)	23 (5.0)	**1.884 (1.141 - 3.111)**	**0.018**	3 (0.3)	70 (6.1)	**16.890 (5.247 - 54.340)**	**<0.0001**

MetS, metabolic syndrome; 95% CI, 95% confidence interval.
[a] Fisher's exact test.

Supplementary Table S5. Combined genotype analysis for the *PAI-1* polymorphisms in ischemic metabolic syndrome patients, stroke patients and controls

Genotype	MetS controls (n=764)	MetS patients (n=235)	OR (95% CI)	*P*	Stroke controls (n=425)	Stroke patients (n=574)	AOR (95% CI)[a]	*P*
***PAI-1* -844G>A/-675 4G>5G**								
GG-4G4G	63 (8.2)	14 (6.0)	1.000 (reference)		38 (8.9)	39 (6.8)	1.000 (reference)	
GG-4G5G	121 (15.8)	34 (14.5)	1.265 (0.632 - 2.528)	0.507	71 (16.7)	84 (14.6)	1.124 (0.625 - 2.024)	0.696
GG-5G5G	104 (13.6)	27 (11.5)	1.168 (0.570 - 2.394)	0.671	53 (12.5)	78 (13.6)	1.328 (0.715 - 2.467)	0.370
GA-4G4G	138 (18.1)	57 (24.3)	1.859 (0.964 - 3.583)	0.064	72 (16.9)	123 (21.4)	1.439 (0.815 - 2.540)	0.209
GA-4G5G	206 (27.0)	62 (26.4)	1.354 (0.711 - 2.581)	0.357	117 (27.5)	151 (26.3)	1.100 (0.646 - 1.872)	0.727
GA-5G5G	12 (1.6)	8 (3.4)	**3.000 (1.034 - 8.709)**	**0.043**	2 (0.5)	18 (3.1)	**8.389 (1.671 - 42.110)**	**0.010**
AA-4G4G	102 (13.4)	26 (11.1)	1.147 (0.557 - 2.361)	0.709	72 (16.9)	56 (9.8)	0.709 (0.377 - 1.334)	0.287
AA-4G5G	12 (1.6)	5 (2.1)	1.875 (0.569 - 6.183)	0.302	0 (0.0)	17 (3.0)	N/A	N/A
AA-5G5G	6 (0.8)	2 (0.9)	1.500 (0.274 - 8.227)	0.641	0 (0.0)	8 (1.4)	N/A	N/A
***PAI-1* -844G>A/43G>A**								
GG-GG	231 (30.2)	56 (23.8)	1.000 (reference)		128 (30.1)	159 (27.7)	1.000 (reference)	
GG-GA	52 (6.8)	18 (7.7)	1.428 (0.776 - 2.629)	0.253	30 (7.1)	40 (7.0)	1.216 (0.693 - 2.135)	0.496
GG-AA	5 (0.7)	1 (0.4)	0.825 (0.095 - 7.202)	0.862	4 (0.9)	2 (0.3)	0.326 (0.054 - 1.962)	0.221
GA-GG	309 (40.4)	114 (48.5)	**1.522 (1.059 - 2.187)**	**0.023**	162 (38.1)	261 (45.5)	1.203 (0.877 - 1.652)	0.252
GA-GA	42 (5.5)	13 (5.5)	1.277 (0.642 - 2.538)	0.486	26 (6.1)	29 (5.1)	0.802 (0.435 - 1.480)	0.480
GA-AA	5 (0.7)	0 (0.0)	0.000 (0.000 - 0.000)	0.995	3 (0.7)	2 (0.3)	0.720 (0.111 - 4.658)	0.730
AA-GG	99 (13.0)	30 (12.8)	1.250 (0.757 - 2.065)	0.384	58 (13.6)	71 (12.4)	1.012 (0.653 - 1.569)	0.958
AA-GA	20 (2.6)	3 (1.3)	0.619 (0.178 - 2.156)	0.451	14 (3.3)	9 (1.6)	0.642 (0.256 - 1.608)	0.344
AA-AA	1 (0.1)	0 (0.0)	N/A	N/A	0 (0.0)	1 (0.2)	N/A	N/A
***PAI-1* -844G>A/9785G>A**								
GG-GG	254 (33.2)	69 (29.4)	1.000 (reference)		147 (34.6)	176 (30.7)	1.000 (reference)	
GG-GA	32 (4.2)	6 (2.6)	0.690 (0.277 - 1.718)	0.425	14 (3.3)	24 (4.2)	1.412 (0.678 - 2.942)	0.357
GG-AA	2 (0.3)	0 (0.0)	0.000 (0.000 - 0.000)	0.994	1 (0.2)	1 (0.2)	N/A	N/A
GA-GG	335 (43.8)	120 (51.1)	1.319 (0.940 - 1.849)	0.109	178 (41.9)	277 (48.3)	1.176 (0.869 - 1.592)	0.293
GA-GA	21 (2.7)	7 (3.0)	1.227 (0.501 - 3.006)	0.654	13 (3.1)	15 (2.6)	0.884 (0.388 - 2.013)	0.768
GA-AA	0 (0.0)	0 (0.0)	N/A	N/A	0 (0.0)	0 (0.0)	N/A	N/A
AA-GG	119 (15.6)	30 (12.8)	0.928 (0.574 - 1.501)	0.761	71 (16.7)	78 (13.6)	0.944 (0.627 - 1.421)	0.782
AA-GA	1 (0.1)	3 (1.3)	**11.044 (1.131 - 107.839)**	**0.039**	1 (0.2)	3 (0.5)	1.718 (0.165 - 17.924)	0.651
AA-AA	0 (0.0)	0 (0.0)	N/A	N/A	0 (0.0)	0 (0.0)	N/A	N/A
***PAI-1* -844G>A/10692T>C**								
GG-TT	70 (9.2)	21 (8.9)	1.000 (reference)		37 (8.7)	54 (9.4)	1.000 (reference)	
GG-TC	112 (14.7)	26 (11.1)	0.774 (0.405 - 1.479)	0.438	67 (15.8)	71 (12.4)	0.789 (0.446 - 1.398)	0.417
GG-CC	106 (13.9)	28 (11.9)	0.881 (0.464 - 1.672)	0.697	58 (13.6)	76 (13.2)	0.898 (0.503 - 1.604)	0.716
GA-TT	103 (13.5)	50 (21.3)	1.618 (0.894 - 2.929)	0.112	64 (15.1)	89 (15.5)	0.802 (0.456 - 1.412)	0.445
GA-TC	236 (30.9)	68 (28.9)	0.961 (0.550 - 1.677)	0.887	123 (28.9)	181 (31.5)	0.964 (0.585 - 1.589)	0.886
GA-CC	17 (2.2)	9 (3.8)	1.765 (0.687 - 4.535)	0.238	4 (0.9)	22 (3.8)	**4.035 (1.145 - 14.217)**	**0.030**
AA-TT	79 (10.3)	21 (8.9)	0.886 (0.447 - 1.758)	0.729	58 (13.6)	42 (7.3)	**0.455 (0.238 - 0.872)**	**0.018**
AA-TC	38 (5.0)	11 (4.7)	0.965 (0.421 - 2.212)	0.933	14 (3.3)	35 (6.1)	**2.659 (1.126 - 6.276)**	**0.026**
AA-CC	3 (0.4)	1 (0.4)	1.111 (0.110 - 11.252)	0.929	0 (0.0)	4 (0.7)	N/A	N/A
***PAI-1* -844G>A/11053T>G**								
GG-TT	147 (19.2)	41 (17.4)	1.000 (reference)		83 (19.5)	105 (18.3)	1.000 (reference)	
GG-TG	103 (13.5)	27 (11.5)	0.940 (0.544 - 1.624)	0.824	66 (15.5)	64 (11.1)	0.791 (0.491 - 1.273)	0.334
GG-GG	38 (5.0)	7 (3.0)	0.661 (0.275 - 1.588)	0.354	13 (3.1)	32 (5.6)	1.896 (0.896 - 4.011)	0.094
GA-TT	50 (6.5)	22 (9.4)	1.578 (0.858 - 2.901)	0.143	24 (5.6)	48 (8.4)	1.284 (0.702 - 2.350)	0.417
GA-TG	234 (30.6)	71 (30.2)	1.088 (0.703 - 1.683)	0.705	128 (30.1)	177 (30.8)	1.059 (0.721 - 1.554)	0.772
GA-GG	72 (9.4)	34 (14.5)	1.693 (0.992 - 2.891)	0.054	39 (9.2)	67 (11.7)	1.123 (0.662 - 1.903)	0.667
AA-TT	9 (1.2)	5 (2.1)	1.992 (0.633 - 6.270)	0.239	1 (0.2)	13 (2.3)	**10.968 (1.351 - 89.059)**	**0.025**
AA-TG	41 (5.4)	8 (3.4)	0.700 (0.304 - 1.609)	0.401	23 (5.4)	26 (4.5)	1.000 (0.509 - 1.964)	0.999
AA-GG	70 (9.2)	20 (8.5)	1.024 (0.559 - 1.877)	0.938	48 (11.3)	42 (7.3)	0.675 (0.393 - 1.159)	0.154

MetS, metabolic syndrome; 95% CI, 95% confidence interval; AOR, adjusted odds ratio; N/A, not applicable.
[a] Adjusted by age, sex, hypertension, diabetes mellitus, hyperlipidemia, and smoking.

Supplementary Table S6. Combined genotype analysis for the *PAI-1* polymorphisms in ischemic metabolic syndrome patients, stroke patients and controls

Genotype	MetS controls (n=764)	MetS patients (n=235)	OR (95% CI)	*P*	Stroke controls (n=425)	Stroke patients (n=574)	AOR (95% CI)[a]	*P*
***PAI-1* -844G>A/12068G>A**								
GG-GG	70 (9.2)	19 (8.1)	1.000 (reference)		37 (8.7)	52 (9.1)	1.000 (reference)	
GG-GA	135 (17.7)	35 (14.9)	0.955 (0.509 - 1.791)	0.886	82 (19.3)	88 (15.3)	0.895 (0.506 - 1.583)	0.703
GG-AA	83 (10.9)	21 (8.9)	0.932 (0.464 - 1.872)	0.843	43 (10.1)	61 (10.6)	1.069 (0.582 - 1.963)	0.831
GA-GG	117 (15.3)	49 (20.9)	1.543 (0.841 - 2.831)	0.161	72 (16.9)	94 (16.4)	0.773 (0.440 - 1.356)	0.369
GA-GA	213 (27.9)	62 (26.4)	1.072 (0.600 - 1.916)	0.814	110 (25.9)	165 (28.7)	1.064 (0.634 - 1.785)	0.814
GA-AA	26 (3.4)	16 (6.8)	**2.267 (1.016 - 5.061)**	**0.046**	9 (2.1)	33 (5.7)	2.316 (0.909 - 5.900)	0.079
AA-GG	78 (10.2)	20 (8.5)	0.945 (0.466 - 1.914)	0.874	54 (12.7)	44 (7.7)	0.540 (0.279 - 1.049)	0.069
AA-GA	35 (4.6)	13 (5.5)	1.368 (0.606 - 3.088)	0.450	18 (4.2)	30 (5.2)	1.498 (0.665 - 3.377)	0.329
AA-AA	7 (0.9)	0 (0.0)	N/A	N/A	0 (0.0)	7 (1.2)	N/A	N/A
***PAI-1* -675 4G>5G/43G>A**								
4G4G-GG	258 (33.8)	94 (40.0)	1.000 (reference)		144 (33.9)	208 (36.2)	1.000 (reference)	
4G4G-GA	44 (5.8)	2 (0.9)	**0.125 (0.030 - 0.525)**	**0.005**	37 (8.7)	9 (1.6)	**0.194 (0.088 - 0.428)**	**0.0001**
4G4G-AA	1 (0.1)	1 (0.4)	2.745 (0.170 - 44.326)	0.477	1 (0.2)	1 (0.2)	0.400 (0.024 - 6.709)	0.524
4G5G-GG	288 (37.7)	79 (33.6)	0.753 (0.534 - 1.061)	0.105	162 (38.1)	205 (35.7)	0.867 (0.636 - 1.180)	0.364
4G5G-GA	44 (5.8)	22 (9.4)	1.372 (0.781 - 2.411)	0.271	22 (5.2)	44 (7.7)	1.312 (0.725 - 2.374)	0.371
4G5G-AA	7 (0.9)	0 (0.0)	N/A	N/A	4 (0.9)	3 (0.5)	0.709 (0.137 - 3.678)	0.683
5G5G-GG	93 (12.2)	27 (11.5)	0.797 (0.489 - 1.300)	0.363	42 (9.9)	78 (13.6)	1.244 (0.786 - 1.968)	0.351
5G5G-GA	26 (3.4)	10 (4.3)	1.056 (0.490 - 2.272)	0.890	11 (2.6)	25 (4.4)	2.071 (0.937 - 4.578)	0.072
5G5G-AA	3 (0.4)	0 (0.0)	N/A	N/A	2 (0.5)	1 (0.2)	0.366 (0.030 - 4.445)	0.430
***PAI-1* -675 4G>5G/9785G>A**								
4G4G-GG	289 (37.8)	91 (38.7)	1.000 (reference)		171 (40.2)	209 (36.4)	1.000 (reference)	
4G4G-GA	14 (1.8)	6 (2.6)	1.361 (0.508 - 3.645)	0.540	11 (2.6)	9 (1.6)	0.530 (0.198 - 1.421)	0.207
4G4G-AA	0 (0.0)	0 (0.0)	N/A	N/A	0 (0.0)	0 (0.0)	N/A	N/A
4G5G-GG	318 (41.6)	97 (41.3)	0.969 (0.698 - 1.344)	0.849	178 (41.9)	237 (41.3)	1.056 (0.787 - 1.417)	0.716
4G5G-GA	21 (2.7)	4 (1.7)	0.605 (0.202 - 1.808)	0.368	10 (2.4)	15 (2.6)	1.148 (0.485 - 2.717)	0.754
4G5G-AA	0 (0.0)	0 (0.0)	N/A	N/A	0 (0.0)	0 (0.0)	N/A	N/A
5G5G-GG	101 (13.2)	31 (13.2)	0.975 (0.612 - 1.554)	0.914	47 (11.1)	85 (14.8)	1.543 (0.999 - 2.384)	0.051
5G5G-GA	19 (2.5)	6 (2.6)	1.003 (0.389 - 2.587)	0.995	7 (1.6)	18 (3.1)	2.221 (0.863 - 5.721)	0.098
5G5G-AA	2 (0.3)	0 (0.0)	N/A	N/A	1 (0.2)	1 (0.2)	N/A	N/A
***PAI-1* -675 4G>5G/10692T>C**								
4G4G-TT	174 (22.8)	64 (27.2)	1.000 (reference)		109 (25.6)	129 (22.5)	1.000 (reference)	
4G4G-TC	109 (14.3)	29 (12.3)	0.723 (0.439 - 1.192)	0.204	63 (14.8)	75 (13.1)	1.200 (0.762 - 1.888)	0.431
4G4G-CC	20 (2.6)	4 (1.7)	0.544 (0.179 - 1.652)	0.283	10 (2.4)	14 (2.4)	1.587 (0.627 - 4.018)	0.330
4G5G-TT	64 (8.4)	23 (9.8)	0.977 (0.560 - 1.704)	0.935	42 (9.9)	45 (7.8)	0.922 (0.546 - 1.558)	0.763
4G5G-TC	234 (30.6)	65 (27.7)	0.755 (0.508 - 1.123)	0.166	123 (28.9)	176 (30.7)	1.268 (0.882 - 1.824)	0.200
4G5G-CC	41 (5.4)	13 (5.5)	0.862 (0.434 - 1.713)	0.672	23 (5.4)	31 (5.4)	1.028 (0.536 - 1.974)	0.933
5G5G-TT	14 (1.8)	5 (2.1)	0.971 (0.336 - 2.804)	0.957	8 (1.9)	11 (1.9)	1.127 (0.397 - 3.200)	0.823
5G5G-TC	43 (5.6)	11 (4.7)	0.696 (0.338 - 1.431)	0.324	18 (4.2)	36 (6.3)	**1.983 (1.011 - 3.891)**	**0.047**
5G5G-CC	65 (8.5)	21 (8.9)	0.878 (0.497 - 1.552)	0.655	29 (6.8)	57 (9.9)	**1.803 (1.030 - 3.154)**	**0.039**
***PAI-1* -675 4G>5G/11053T>G**								
4G4G-TT	42 (5.5)	14 (6.0)	1.000 (reference)		24 (5.6)	32 (5.6)	1.000 (reference)	
4G4G-TG	123 (16.1)	31 (13.2)	0.756 (0.367 - 1.556)	0.448	80 (18.8)	74 (12.9)	0.758 (0.388 - 1.479)	0.416
4G4G-GG	138 (18.1)	52 (22.1)	1.130 (0.571 - 2.240)	0.725	78 (18.4)	112 (19.5)	0.944 (0.494 - 1.807)	0.863
4G5G-TT	74 (9.7)	25 (10.6)	1.014 (0.476 - 2.158)	0.972	44 (10.4)	55 (9.6)	0.884 (0.438 - 1.784)	0.730
4G5G-TG	227 (29.7)	67 (28.5)	0.886 (0.456 - 1.719)	0.719	124 (29.2)	170 (29.6)	0.960 (0.526 - 1.754)	0.895
4G5G-GG	38 (5.0)	9 (3.8)	0.711 (0.276 - 1.829)	0.479	20 (4.7)	27 (4.7)	1.047 (0.445 - 2.465)	0.917
5G5G-TT	90 (11.8)	29 (12.3)	0.967 (0.463 - 2.017)	0.928	40 (9.4)	79 (13.8)	1.552 (0.754 - 3.194)	0.233
5G5G-TG	28 (3.7)	8 (3.4)	0.857 (0.318 - 2.311)	0.761	13 (3.1)	23 (4.0)	1.267 (0.483 - 3.326)	0.630
5G5G-GG	4 (0.5)	0 (0.0)	N/A	N/A	2 (0.5)	2 (0.3)	0.552 (0.060 - 5.083)	0.600

MetS, metabolic syndrome; 95% CI, 95% confidence interval; AOR, adjusted odds ratio; N/A, not applicable.

[a] Adjusted by age, sex, hypertension, diabetes mellitus, hyperlipidemia, and smoking.

Supplementary Table S7. Combined genotype analysis for the *PAI-1* polymorphisms in ischemic metabolic syndrome patients, stroke patients and controls

Genotype	MetS controls (n=764)	MetS patients (n=235)	OR (95% CI)	*P*	Stroke controls (n=425)	Stroke patients (n=574)	AOR (95% CI)[a]	*P*
***PAI-1* -675 4G>5G/12068G>A**								
4G4G-GG	162 (21.2)	61 (26.0)	1.000 (reference)		101 (23.8)	122 (21.3)	1.000 (reference)	
4G4G-GA	119 (15.6)	30 (12.8)	0.670 (0.407 - 1.101)	0.114	71 (16.7)	78 (13.6)	1.107 (0.707 - 1.733)	0.657
4G4G-AA	22 (2.9)	6 (2.6)	0.724 (0.280 - 1.872)	0.506	10 (2.4)	18 (3.1)	1.690 (0.699 - 4.084)	0.244
4G5G-GG	86 (11.3)	21 (8.9)	0.649 (0.370 - 1.136)	0.130	56 (13.2)	51 (8.9)	0.729 (0.445 - 1.194)	0.210
4G5G-GA	206 (27.0)	63 (26.8)	0.812 (0.540 - 1.221)	0.317	110 (25.9)	159 (27.7)	1.343 (0.913 - 1.975)	0.134
4G5G-AA	47 (6.2)	17 (7.2)	0.961 (0.513 - 1.800)	0.900	22 (5.2)	42 (7.3)	1.640 (0.880 - 3.057)	0.120
5G5G-GG	17 (2.2)	6 (2.6)	0.937 (0.353 - 2.488)	0.897	6 (1.4)	17 (3.0)	2.430 (0.852 - 6.931)	0.097
5G5G-GA	58 (7.6)	17 (7.2)	0.778 (0.421 - 1.441)	0.425	29 (6.8)	46 (8.0)	1.637 (0.916 - 2.927)	0.096
5G5G-AA	47 (6.2)	14 (6.0)	0.791 (0.407 - 1.539)	0.490	20 (4.7)	41 (7.1)	**1.908 (1.002 - 3.631)**	**0.049**
***PAI-1* 43G>A/9785G>A**								
GG-GG	591 (77.4)	185 (78.7)	1.000 (reference)		325 (76.5)	451 (78.6)	1.000 (reference)	
GG-GA	46 (6.0)	15 (6.4)	1.042 (0.568 - 1.909)	0.895	22 (5.2)	39 (6.8)	1.177 (0.671 - 2.066)	0.569
GG-AA	2 (0.3)	0 (0.0)	N/A	N/A	1 (0.2)	1 (0.2)	N/A	N/A
GA-GG	106 (13.9)	33 (14.0)	0.995 (0.651 - 1.520)	0.980	64 (15.1)	75 (13.1)	0.862 (0.589 - 1.262)	0.446
GA-GA	8 (1.0)	1 (0.4)	0.399 (0.050 - 3.214)	0.388	6 (1.4)	3 (0.5)	0.465 (0.110 - 1.958)	0.296
GA-AA	0 (0.0)	0 (0.0)	N/A	N/A	0 (0.0)	0 (0.0)	N/A	N/A
AA-GG	11 (1.4)	1 (0.4)	0.290 (0.037 - 2.265)	0.238	7 (1.6)	5 (0.9)	0.556 (0.167 - 1.847)	0.338
AA-GA	0 (0.0)	0 (0.0)	N/A	N/A	0 (0.0)	0 (0.0)	N/A	N/A
AA-AA	0 (0.0)	0 (0.0)	N/A	N/A	0 (0.0)	0 (0.0)	N/A	N/A
***PAI-1* 43G>A/10692T>C**								
GG-TT	219 (28.7)	85 (36.2)	1.000 (reference)		130 (30.6)	174 (30.3)	1.000 (reference)	
GG-TC	316 (41.4)	86 (36.6)	**0.701 (0.496 - 0.991)**	**0.044**	167 (39.3)	235 (40.9)	1.126 (0.821 - 1.544)	0.463
GG-CC	104 (13.6)	29 (12.3)	0.718 (0.444 - 1.163)	0.179	51 (12.0)	82 (14.3)	1.289 (0.830 - 2.002)	0.259
GA-TT	32 (4.2)	7 (3.0)	0.564 (0.240 - 1.326)	0.189	29 (6.8)	10 (1.7)	**0.269 (0.120 - 0.604)**	**0.001**
GA-TC	65 (8.5)	18 (7.7)	0.714 (0.400 - 1.273)	0.253	33 (7.8)	50 (8.7)	1.302 (0.770 - 2.202)	0.325
GA-CC	17 (2.2)	9 (3.8)	1.364 (0.585 - 3.178)	0.472	8 (1.9)	18 (3.1)	1.773 (0.710 - 4.430)	0.220
AA-TT	1 (0.1)	0 (0.0)	N/A	N/A	0 (0.0)	1 (0.2)	N/A	N/A
AA-TC	5 (0.7)	1 (0.4)	0.515 (0.059 - 4.476)	0.548	4 (0.9)	2 (0.3)	0.445 (0.075 - 2.635)	0.372
AA-CC	5 (0.7)	0 (0.0)	N/A	N/A	3 (0.7)	2 (0.3)	0.517 (0.076 - 3.502)	0.499
***PAI-1* 43G>A/11053T>G**								
GG-TT	166 (21.7)	53 (22.6)	1.000 (reference)		87 (20.5)	132 (23.0)	1.000 (reference)	
GG-TG	313 (41.0)	87 (37.0)	0.871 (0.590 - 1.286)	0.486	177 (41.6)	223 (38.9)	0.866 (0.612 - 1.224)	0.415
GG-GG	160 (20.9)	60 (25.5)	1.175 (0.765 - 1.803)	0.462	84 (19.8)	136 (23.7)	1.042 (0.695 - 1.562)	0.842
GA-TT	34 (4.5)	15 (6.4)	1.382 (0.699 - 2.732)	0.353	18 (4.2)	31 (5.4)	1.225 (0.625 - 2.402)	0.554
GA-TG	60 (7.9)	18 (7.7)	0.940 (0.510 - 1.731)	0.842	36 (8.5)	42 (7.3)	0.800 (0.461 - 1.387)	0.426
GA-GG	20 (2.6)	1 (0.4)	0.157 (0.021 - 1.195)	0.074	16 (3.8)	5 (0.9)	**0.247 (0.085 - 0.712)**	**0.010**
AA-TT	6 (0.8)	0 (0.0)	N/A	N/A	3 (0.7)	3 (0.5)	0.690 (0.127 - 3.761)	0.668
AA-TG	5 (0.7)	1 (0.4)	0.626 (0.072 - 5.482)	0.673	4 (0.9)	2 (0.3)	0.366 (0.062 - 2.167)	0.268
AA-GG	0 (0.0)	0 (0.0)	N/A	N/A	0 (0.0)	0 (0.0)	N/A	N/A
***PAI-1* 43G>A/12068G>A**								
GG-GG	232 (30.4)	82 (34.9)	1.000 (reference)		134 (31.5)	180 (31.4)	1.000 (reference)	
GG-GA	314 (41.1)	90 (38.3)	0.811 (0.575 - 1.144)	0.232	169 (39.8)	235 (40.9)	1.164 (0.849 - 1.595)	0.347
GG-AA	93 (12.2)	28 (11.9)	0.852 (0.521 - 1.393)	0.523	45 (10.6)	76 (13.2)	1.329 (0.843 - 2.094)	0.221
GA-GG	31 (4.1)	5 (2.1)	0.456 (0.172 - 1.213)	0.116	28 (6.6)	8 (1.4)	**0.215 (0.092 - 0.502)**	**0.0003**
GA-GA	63 (8.2)	20 (8.5)	0.898 (0.512 - 1.576)	0.708	36 (8.5)	47 (8.2)	1.200 (0.700 - 2.055)	0.507
GA-AA	20 (2.6)	9 (3.8)	1.273 (0.557 - 2.908)	0.567	6 (1.4)	23 (4.0)	**3.184 (1.195 - 8.483)**	**0.021**
AA-GG	2 (0.3)	1 (0.4)	1.415 (0.127 - 15.809)	0.778	1 (0.2)	2 (0.3)	0.970 (0.085 - 11.074)	0.981
AA-GA	6 (0.8)	0 (0.0)	N/A	N/A	5 (1.2)	1 (0.2)	0.230 (0.025 - 2.102)	0.193
AA-AA	3 (0.4)	0 (0.0)	N/A	N/A	1 (0.2)	2 (0.3)	1.772 (0.135 - 23.293)	0.663

MetS, metabolic syndrome; 95% CI, 95% confidence interval; AOR, adjusted odds ratio; N/A, not applicable.
[a] Adjusted by age, sex, hypertension, diabetes mellitus, hyperlipidemia, and smoking.

Supplementary Table S8. Combined genotype analysis for the *PAI-1* polymorphisms in ischemic metabolic syndrome patients, stroke patients and controls

Genotype	MetS controls (n=764)	MetS patients (n=235)	OR (95% CI)	*P*	Stroke controls (n=425)	Stroke patients (n=574)	AOR (95% CI)[a]	*P*
***PAI-1* 9785G>A/10692T>C**								
GG-TT	241 (31.5)	86 (36.6)	1.000 (reference)		152 (35.8)	175 (30.5)	1.000 (reference)	
GG-TC	360 (47.1)	100 (42.6)	0.778 (0.559 - 1.084)	0.138	190 (44.7)	270 (47.0)	1.335 (0.989 - 1.803)	0.059
GG-CC	107 (14.0)	33 (14.0)	0.864 (0.545 - 1.371)	0.536	54 (12.7)	86 (15.0)	1.452 (0.947 - 2.226)	0.087
GA-TT	11 (1.4)	6 (2.6)	1.529 (0.549 - 4.259)	0.417	7 (1.6)	10 (1.7)	1.072 (0.376 - 3.062)	0.896
GA-TC	26 (3.4)	5 (2.1)	0.539 (0.201 - 1.448)	0.220	14 (3.3)	17 (3.0)	1.103 (0.507 - 2.401)	0.805
GA-CC	17 (2.2)	5 (2.1)	0.824 (0.295 - 2.302)	0.712	7 (1.6)	15 (2.6)	2.259 (0.850 - 6.004)	0.102
AA-TT	0 (0.0)	0 (0.0)	N/A	N/A	0 (0.0)	0 (0.0)	N/A	N/A
AA-TC	0 (0.0)	0 (0.0)	N/A	N/A	0 (0.0)	0 (0.0)	N/A	N/A
AA-CC	2 (0.3)	0 (0.0)	N/A	N/A	1 (0.2)	1 (0.2)	N/A	N/A
***PAI-1* 9785G>A/11053T>G**								
GG-TT	176 (23.0)	61 (26.0)	1.000 (reference)		94 (22.1)	143 (24.9)	1.000 (reference)	
GG-TG	355 (46.5)	99 (42.1)	0.805 (0.558 - 1.161)	0.245	205 (48.2)	249 (43.4)	0.827 (0.594 - 1.152)	0.262
GG-GG	177 (23.2)	59 (25.1)	0.962 (0.636 - 1.455)	0.854	97 (22.8)	139 (24.2)	0.915 (0.622 - 1.347)	0.653
GA-TT	28 (3.7)	7 (3.0)	0.721 (0.300 - 1.736)	0.466	13 (3.1)	22 (3.8)	1.210 (0.559 - 2.616)	0.629
GA-TG	23 (3.0)	7 (3.0)	0.878 (0.359 - 2.149)	0.776	12 (2.8)	18 (3.1)	0.952 (0.421 - 2.150)	0.905
GA-GG	3 (0.4)	2 (0.9)	1.924 (0.314 - 11.786)	0.479	3 (0.7)	2 (0.3)	0.344 (0.053 - 2.227)	0.263
AA-TT	2 (0.3)	0 (0.0)	N/A	N/A	1 (0.2)	1 (0.2)	N/A	N/A
AA-TG	0 (0.0)	0 (0.0)	N/A	N/A	0 (0.0)	0 (0.0)	N/A	N/A
AA-GG	0 (0.0)	0 (0.0)	N/A	N/A	0 (0.0)	0 (0.0)	N/A	N/A
***PAI-1* 9785G>A/12068G>A**								
GG-GG	237 (31.0)	78 (33.2)	1.000 (reference)		151 (35.5)	164 (28.6)	1.000 (reference)	
GG-GA	357 (46.7)	104 (44.3)	0.885 (0.632 - 1.239)	0.477	195 (45.9)	266 (46.3)	**1.415 (1.041 - 1.923)**	**0.027**
GG-AA	114 (14.9)	37 (15.7)	0.986 (0.629 - 1.548)	0.952	50 (11.8)	101 (17.6)	**1.944 (1.270 - 2.974)**	**0.002**
GA-GG	27 (3.5)	10 (4.3)	1.125 (0.521 - 2.429)	0.764	12 (2.8)	25 (4.4)	1.766 (0.828 - 3.768)	0.141
GA-GA	25 (3.3)	6 (2.6)	0.729 (0.289 - 1.843)	0.504	14 (3.3)	17 (3.0)	1.323 (0.598 - 2.926)	0.490
GA-AA	2 (0.3)	0 (0.0)	N/A	N/A	2 (0.5)	0 (0.0)	N/A	N/A
AA-GG	1 (0.1)	0 (0.0)	N/A	N/A	0 (0.0)	1 (0.2)	N/A	N/A
AA-GA	1 (0.1)	0 (0.0)	N/A	N/A	1 (0.2)	0 (0.0)	N/A	N/A
AA-AA	0 (0.0)	0 (0.0)	N/A	N/A	0 (0.0)	0 (0.0)	N/A	N/A
***PAI-1* 10692T>C/11053T>G**								
TT-TT	28 (3.7)	17 (7.2)	1.000 (reference)		19 (4.5)	26 (4.5)	1.000 (reference)	
TT-TG	59 (7.7)	18 (7.7)	0.503 (0.226 - 1.120)	0.092	45 (10.6)	32 (5.6)	0.580 (0.255 - 1.317)	0.193
TT-GG	165 (21.6)	57 (24.3)	0.569 (0.290 - 1.116)	0.101	95 (22.4)	127 (22.1)	1.067 (0.532 - 2.140)	0.855
TC-TT	59 (7.7)	16 (6.8)	0.447 (0.197 - 1.012)	0.053	30 (7.1)	45 (7.8)	1.335 (0.594 - 3.003)	0.485
TC-TG	315 (41.2)	85 (36.2)	**0.444 (0.232 - 0.850)**	**0.014**	170 (40.0)	230 (40.1)	1.165 (0.610 - 2.226)	0.644
TC-GG	12 (1.6)	4 (1.7)	0.549 (0.152 - 1.979)	0.359	4 (0.9)	12 (2.1)	1.890 (0.456 - 7.830)	0.380
CC-TT	119 (15.6)	35 (14.9)	**0.484 (0.238 - 0.986)**	**0.046**	59 (13.9)	95 (16.6)	1.381 (0.665 - 2.872)	0.387
CC-TG	4 (0.5)	3 (1.3)	1.235 (0.246 - 6.203)	0.797	2 (0.5)	5 (0.9)	4.567 (0.571 - 36.526)	0.152
CC-GG	3 (0.4)	0 (0.0)	0.000 (0.000 - 0.000)	0.994	1 (0.2)	2 (0.3)	6.053 (0.135 - 272.385)	0.354
***PAI-1* 10692T>C/12068G>A**								
TT-GG	186 (24.3)	66 (28.1)	1.000 (reference)		120 (28.2)	132 (23.0)	1.000 (reference)	
TT-GA	60 (7.9)	21 (8.9)	0.986 (0.557 - 1.746)	0.962	37 (8.7)	44 (7.7)	1.255 (0.736 - 2.139)	0.405
TT-AA	6 (0.8)	5 (2.1)	2.349 (0.694 - 7.952)	0.170	2 (0.5)	9 (1.6)	4.327 (0.842 - 22.235)	0.079
TC-GG	74 (9.7)	17 (7.2)	0.647 (0.356 - 1.177)	0.154	42 (9.9)	49 (8.5)	1.081 (0.651 - 1.797)	0.763
TC-GA	283 (37.0)	79 (33.6)	0.787 (0.540 - 1.145)	0.211	150 (35.3)	212 (36.9)	**1.485 (1.051 - 2.098)**	**0.025**
TC-AA	29 (3.8)	9 (3.8)	0.875 (0.393 - 1.944)	0.742	12 (2.8)	26 (4.5)	2.143 (0.996 - 4.609)	0.051
CC-GG	5 (0.7)	5 (2.1)	2.818 (0.791 - 10.045)	0.110	1 (0.2)	9 (1.6)	6.321 (0.709 - 56.389)	0.099
CC-GA	40 (5.2)	10 (4.3)	0.705 (0.334 - 1.488)	0.359	23 (5.4)	27 (4.7)	1.283 (0.665 - 2.474)	0.457
CC-AA	81 (10.6)	23 (9.8)	0.800 (0.466 - 1.375)	0.420	38 (8.9)	66 (11.5)	**1.707 (1.039 - 2.804)**	**0.035**

MetS, metabolic syndrome; 95% CI, 95% confidence interval; AOR, adjusted odds ratio; N/A, not applicable.

[a] Adjusted by age, sex, hypertension, diabetes mellitus, hyperlipidemia, and smoking.

Supplementary Table S9. Clinical variables in ischemic stroke patients stratified by *PAI-1* polymorphisms status by ANOVA

Characteristics	Homocysteine (mmol/L)		Folate (mg/ml)		Vitamin B12 (pg/ml)		Total cholesterol (mg/dl)		Triglyceride (mg/dl)		PLT (10^3/μℓ)		BMI (kg/m^2)	
	Mean ± SD	P[a]	Mean ± SD	P[a]	Mean ± SD	P[a]	Mean ± SD	P[a]	Mean ± SD	P[a]	Mean ± SD	P[a]	Mean ± SD	P[a]
***PAI-1* -844G>A**														
GG	10.11±4.00	0.080	7.71±5.58	0.943	745.42±718.65	0.985	190.29±37.86	0.158[b]	151.21±104.46	0.619	244.02±86.77	0.578	24.14±3.16	0.744
GA	11.01±6.68		7.86±7.44		739.71±604.99		193.40±39.02		149.89±95.69		247.21±71.30		24.14±3.04	
AA	10.75±6.32		7.83±5.51		735.34±543.02		194.97±43.02		142.26±75.53		252.00±86.54		24.39±3.45	
Dominant (GG vs GA+AA)	10.95±6.59	0.165[c]	7.85±7.02	0.595[c]	738.66±590.24	0.294[c]	193.78±39.98	0.180	148.06±91.27	0.807[c]	248.36±75.22	0.190[c]	24.20±3.14	0.788
Recessive (GG+GA vs AA)	10.75±6.32	0.802	7.83±5.51	0.797[c]	735.34±543.02	0.481c	194.97±43.02	0.404	142.26±75.53	0.958[c]	252.00±86.54	0.381	24.39±3.45	0.442
***PAI-1* -675 4G>5G**														
4G4G	10.46±4.85	0.347	7.59±4.68	0.380	720.68±484.36	0.578	194.91±40.53	0.136	145.12±92.71	0.213	252.38±91.82	0.184	24.13±3.01	0.787
4G5G	10.93±6.58		8.20±8.29		764.90±728.88		192.14±39.57		155.19±104.99		243.97±72.59		24.06±3.15	
5G5G	10.28±5.61		7.21±4.56		725.25±708.50		187.56±34.49		142.74±76.93		240.77±63.48		24.57±3.41	
Dominant (4G4G vs 4G5G+5G5G)	10.76±6.34	0.587[c]	7.94±7.51	0.380[c]	754.51±723.21	0.120	190.93±38.32	0.279	151.90±98.46	0.124[c]	243.12±70.25	0.067[c]	24.20±3.23	0.756
Recessive (4G4G+4G5G vs 5G5G)	10.28±5.61	0.393	7.21±4.56	0.405[c]	725.25±708.50	**0.023**[c]	187.56±34.49	0.152[c]	142.74±76.93	0.979[c]	240.77±63.48	0.398[c]	24.57±3.41	0.108
***PAI-1* 43G>A**														
GG	10.79±6.11	0.145	7.83±6.72	0.924	751.71±681.13	0.467	192.45±38.75	0.988	149.53±98.84	0.599	246.90±81.45	0.909	24.11±3.08	0.264
GA	9.86±3.63		7.60±5.57		688.67±337.51		192.95±42.37		149.62±83.13		245.43±70.05		24.62±3.49	
AA	9.27±2.35		7.98±4.05		630.82±272.70		191.75±37.04		121.25±41.42		255.58±57.15		23.49±3.13	
Dominant (GG vs GA+AA)	9.82±3.55	0.258[c]	7.63±5.47	0.777[c]	684.56±332.91	0.446[c]	192.86±41.87	0.905	147.41±80.93	0.663[c]	246.21±69.04	0.846[c]	24.54±3.47	0.175
Recessive (GG+GA vs AA)	9.27±2.35	0.554[c]	7.98±4.05	0.926	630.82±272.70	0.385[c]	191.75±37.04	0.946	121.25±41.42	0.579[c]	255.58±57.15	0.700	23.49±3.13	0.534
***PAI-1* 9785G>A**														
GG	10.69±5.95	0.577	7.80±6.63	0.996	739.95±650.83	0.903	192.52±39.14	0.398	149.09±96.97	0.991	246.44±80.94	0.789	24.18±3.11	0.960
GA	9.95±3.11		7.86±5.23		760.81±477.19		191.29±40.75		150.69±87.51		252.04±60.45		24.16±3.67	
AA	10.07±0.98		7.61±0.47		574.50±74.25		229.50±23.33		150.00±73.54		224.50±10.61		22.00±0.00	
Dominant (GG vs GA+AA)	9.95±3.07	0.784[c]	7.86±5.15	0.508[c]	755.64±471.52	0.682[c]	192.35±40.76	0.971	150.67±86.70	0.893	251.27±59.77	0.199[c]	24.12±3.65	0.893
Recessive (GG+GA vs AA)	10.07±0.98	0.889	7.61±0.47	0.967	574.50±74.25	0.712	229.50±23.33	0.182	150.00±73.54	0.991	224.50±10.61	0.692	22.00±0.00	0.489
***PAI-1* 10692T>C**														
TT	10.74±4.96	0.781	7.93±5.70	0.908	750.07±630.89	0.320	192.09±39.85	0.962	147.54±101.25	0.775	246.07±93.49	0.962	23.97±2.96	0.245
TC	10.51±5.53		7.73±7.05		700.84±401.96		192.61±39.23		151.38±97.10		246.83±71.18		24.32±3.20	
CC	10.81±7.82		7.75±6.61		843.40±1079.74		193.09±38.22		146.16±82.23		248.17±71.96		24.17±3.35	
Dominant (TT vs TC+CC)	10.59±6.18	0.250[c]	7.74±6.93	0.368[c]	736.42±643.93	0.751	192.73±38.95	0.808	150.07±93.55	0.696	247.17±71.32	0.582[c]	24.28±3.23	0.196
Recessive (TT+TC vs CC)	10.81±7.82	0.947[c]	7.75±6.61	0.922	843.40±1079.74	0.265[c]	193.09±38.22	0.838	146.16±82.23	0.782[c]	248.17±71.96	0.809	24.17±3.35	0.977
***PAI-1* 11053T>G**														
TT	10.28±6.27	0.483	7.90±8.39	0.949	796.25±892.43	0.667	195.19±37.60	0.217	149.13±99.31	0.935	247.57±73.55	0.661	24.17±3.25	0.944
TG	10.76±5.80		7.79±5.96		707.22±409.48		190.30±39.89		148.30±95.36		242.79±67.54		24.29±3.15	
GG	10.80±5.17		7.71±5.10		746.74±672.34		193.88±39.68		151.10±94.72		253.99±104.63		23.95±3.02	
Dominant (TT vs TG+GG)	10.78±5.60	0.358[c]	7.77±5.69	0.611[c]	720.26±511.25	0.667[c]	191.49±39.83	0.186	149.23±95.09	0.989	246.50±81.80	0.661[c]	24.18±3.11	0.967
Recessive (TT+TG vs GG)	10.80±5.17	0.513[c]	7.71±5.10	0.775[c]	746.74±672.34	0.877	193.88±39.68	0.539	151.10±94.72	0.728	253.99±104.63	0.176[c]	23.95±3.02	0.256
***PAI-1* 12068G>A**														
GG	10.64±4.53	0.827	8.10±7.72	0.272	743.00±601.25	0.422	192.50±40.95	0.998	146.90±87.05	0.564	254.80±100.90	0.112	24.11±3.24	0.786
GA	10.72±6.92		7.46±5.24		723.55±515.79		192.46±38.23		152.41±106.50		241.19±63.11		24.27±3.08	
AA	10.39±4.20		8.21±7.28		794.40±995.98		192.70±38.66		144.18±80.43		246.28±69.45		24.02±3.17	
Dominant (GG vs GA+AA)	10.64±6.38	0.150[c]	7.64±5.78	0.413[c]	740.06±659.42	0.945	192.52±38.30	0.994	150.47±100.94	0.799[c]	242.39±64.64	0.112[c]	24.21±3.10	0.663
Recessive (GG+GA vs AA)	10.39±4.20	0.880[c]	8.21±7.28	0.764[c]	794.40±995.98	0.244[c]	192.70±38.66	0.949	144.18±80.43	0.959[c]	246.28±69.45	0.666[c]	24.02±3.17	0.544

ANOVA, analysis of variance; PLT, platelet; SD, standard deviation.
[a] Calculated using ANOVA. [b] Calculated using the Kruskal-Wallis test. [c] Calculated using the Mann-Whitney test.

Supplementary Table S10. Clinical variables in ischemic stroke patients stratified by *PAI-1* polymorphisms status by ANOVA.

Characteristics	PT (sec)		aPTT (sec)		Fibrinogen (mg/dl)		Antithrombin III (%)		BUN (mg/dl)		Uric Acid (mg/dl)		HDL-cholsterol (mg/dl)	
	Mean ± SD	*P*[a]	Mean ± SD	*P*[a]	Mean ± SD	*P*[a]	Mean ± SD	*P*[a]	Mean ± SD	*P*[a]	Mean ± SD	*P*[a]	Mean ± SD	*P*[a]
***PAI-1* -844G>A**														
GG	11.82±1.17	0.917[b]	31.69±8.67	0.628	409.98±135.24	0.495	95.28±35.55	0.506	15.78±5.29	0.819	4.61±1.51	0.609	45.30±14.91	0.915
GA	11.75±0.72		31.56±14.31		421.89±125.09		93.10±18.95		15.99±7.04		4.70±1.58		45.31±15.90	
AA	11.74±0.82		30.56±5.22		410.58±115.38		95.82±15.47		16.13±7.02		4.58±1.35		46.40±11.57	
Dominant (GG vs GA+AA)	11.75±0.74	0.917[c]	31.33±12.81	0.249[c]	419.40±122.97	0.360	93.70±18.26	0.881[c]	16.03±7.03	0.695[c]	4.67±1.53	0.578	45.56±15.01	0.823
Recessive (GG+GA vs AA)	11.74±0.82	0.598	30.56±5.22	0.360[c]	410.58±115.38	0.662	95.82±15.47	0.165[c]	16.13±7.02	0.684	4.58±1.35	0.704[c]	46.40±11.57	0.489
***PAI-1* -675 4G>5G**														
4G4G	11.75±0.82	0.519	30.87±6.87	0.479	421.32±131.07	0.626	93.34±16.71	0.081	15.72±4.92	0.497	4.53±1.47	0.072	45.27±13.44	0.390
4G5G	11.82±1.03		31.82±14.95		414.43±123.28		93.22±20.10		16.21±7.87		4.77±1.57		45.57±16.58	
5G5G	11.74±0.83		31.90±9.01		407.88±131.38		99.30±47.89		15.71±5.40		4.60±1.51		45.65±13.86	
Dominant (4G4G vs 4G5G+5G5G)	11.80±0.98	0.495[c]	31.84±13.58	0.172[c]	412.64±125.41	0.395	94.86±30.28	0.904[c]	16.08±7.30	0.993[c]	4.73±1.55	0.050	45.59±15.87	0.780
Recessive (4G4G+4G5G vs 5G5G)	11.74±0.83	0.602	31.90±9.01	0.208[c]	407.88±131.38	0.463	99.30±47.89	0.119[c]	15.71±5.40	0.723[c]	4.60±1.51	0.686	45.65±13.86	0.882
***PAI-1* 43G>A**														
GG	11.79±0.93	0.134	31.62±12.32	0.656	416.97±128.49	0.611	94.31±27.23	0.943	16.03±6.77	0.547	4.66±1.52	0.755	45.40±13.78	0.052
GA	11.68±0.87		30.62±3.86		406.90±121.80		93.95±17.48		15.43±4.38		4.59±1.55		45.81±21.48	
AA	12.26±0.93		30.40±3.26		450.00±134.83		97.67±11.74		15.35±4.75		4.42±1.30		47.02±9.98	
Dominant (GG vs GA+AA)	11.73±0.88	0.501	30.61±3.81	0.595[c]	410.01±122.54	0.620	94.18±17.15	0.423[c]	15.42±4.39	0.725[c]	4.58±1.53	0.517	45.87±21.01	0.771
Recessive (GG+GA vs AA)	12.26±0.93	0.099	30.40±3.26	0.904[c]	450.00±134.83	0.478	97.67±11.74	0.749	15.35±4.75	0.752	4.42±1.30	0.594	47.02±9.98	0.816
***PAI-1* 9785G>A**														
GG	11.78±0.93	0.873	31.20±7.19	0.465[b]	413.25±125.75	0.050	93.89±25.92	0.158	15.97±6.56	0.838	4.66±1.53	0.772	45.48±15.26	0.471
GA	11.75±0.88		35.01±34.80		450.74±146.07		99.46±26.88		15.49±5.07		4.52±1.40		45.25±10.56	
AA	11.35±0.00		33.10±0.00		0.00±0.00		0.00±0.00		16.20±3.11		4.75±0.07		0.00±0.00	
Dominant (GG vs GA+AA)	11.74±0.87	0.767	34.98±34.51	0.465[c]	450.74±146.07	0.050	99.46±26.88	0.158	15.51±5.01	0.568[c]	4.53±1.38	0.490	45.25±10.56	0.915
Recessive (GG+GA vs AA)	11.35±0.00	N/A	33.10±0.00	N/A	0.00±0.00	N/A	0.00±0.00	N/A	16.20±3.11	0.954	4.75±0.07	0.926	0.00±0.00	
***PAI-1* 10692T>C**														
TT	11.74±0.78	**0.029**	30.70±6.59	0.366	413.98±120.09	0.957	92.35±17.04	0.310	15.88±5.10	0.338	4.73±1.48	0.461	43.95±11.08	0.116
TC	11.85±1.01		31.81±14.44		417.23±132.87		94.76±32.01		15.75±6.01		4.61±1.50		46.83±17.90	
CC	11.63±0.91		32.02±8.91		416.04±126.61		96.84±18.49		16.60±9.54		4.60±1.66		44.52±11.73	
Dominant (TT vs TC+CC)	11.80±0.99	0.424[c]	31.86±13.29	0.079[c]	416.95±131.28	0.777	95.24±29.41	0.325[c]	15.96±7.07	0.646[c]	4.61±1.54	0.215	46.26±16.60	**0.050**
Recessive (TT+TC vs CC)	11.63±0.91	**0.039**	32.02±8.91	0.074[c]	416.04±126.61	0.994	96.84±18.49	0.111[c]	16.60±9.54	0.550[c]	4.60±1.66	0.624	44.52±11.73	0.457
***PAI-1* 11053T>G**														
TT	11.68±0.83	0.085	32.06±8.62	0.235	409.59±129.51	0.527	94.32±18.05	0.872	15.74±4.76	0.755	4.62±1.48	0.930	44.18±11.62	0.670
TG	11.84±1.01		31.71±14.49		415.04±128.41		94.71±32.86		16.09±7.72		4.65±1.53		46.70±18.18	
GG	11.75±0.83		30.32±6.29		424.94±123.96		93.39±16.34		15.85±5.26		4.67±1.55		44.63±11.23	
Dominant (TT vs TG+GG)	11.81±0.95	**0.025[c]**	31.24±12.36	**0.013[c]**	418.39±126.88	0.426	94.28±28.48	0.435[c]	16.01±6.99	0.674[c]	4.66±1.54	0.729	45.97±16.07	0.152
Recessive (TT+TG vs GG)	11.75±0.83	0.625[c]	30.32±6.29	**0.013[c]**	424.94±123.96	0.301	93.39±16.34	0.879[c]	15.85±5.26	0.939[c]	4.67±1.55	0.789	44.63±11.23	0.387
***PAI-1* 12068G>A**														
GG	11.77±0.79	0.985	31.54±16.44	0.835	425.24±121.06	0.329	93.07±20.12	0.676	15.89±5.08	0.745	4.65±1.51	0.949	44.98±11.12	0.350
GA	11.78±1.02		31.27±7.83		409.00±124.16		94.82±31.06		15.85±6.12		4.64±1.46		46.26±18.11	
AA	11.78±0.89		31.95±6.12		417.80±150.53		95.24±18.87		16.31±9.63		4.69±1.72		44.17±11.58	
Dominant (GG vs GA+AA)	11.78±0.99	0.800[c]	31.43±7.47	**0.039[c]**	411.08±130.74	0.174	94.92±28.59	0.491[c]	15.96±7.10	0.429[c]	4.65±1.53	0.973	45.73±16.73	0.517
Recessive (GG+GA vs AA)	11.78±0.89	0.917	31.95±6.12	**0.004[c]**	417.80±150.53	0.575[c]	95.24±18.87	0.658[c]	16.31±9.63	0.962[c]	4.69±1.72	0.690[c]	44.17±11.58	0.311

ANOVA, analysis of variance; PT, prothrombin time; aPTT, activated partial thromboplastin time; SD, standard deviation; N/A, not applicable.
[a] Calculated using ANOVA. [b] Calculated using the Kruskal-Wallis test. [c] Calculated using the Mann-Whitney test.

Supplementary Table S11. Baseline characteristics between ischemic stroke patients and control subjects in sample 1 and 2

Characteristic	Sample 1*			Sample2**		
	Controls (n=259)	Stroke patients (n=205)	P^*	Controls (n=166)	Stroke patients (n=369)	P^*
	259	205		166	369	
Male (%)	95 (36.7)	84 (41.0)	0.3456	78 (47.0)	150 (40.7)	0.171
Age (years, mean ±SD)	62.9±10.5	62.1±10.7	0.376	62.2±11.2	62.9±12.0	0.713
Smoking (%)	72 (27.8)	71 (35.7)	0.072	66 (39.8)	135 (36.7)	0.498
Metabolic syndrome (%)	29 (11.2)	67 (32.7)	< 0.0001	19 (11.4)	120 (32.5)	< 0.0001
Hypertension (%)	107 (41.3)	124 (60.5)	< 0.0001	63 (38.0)	238 (64.5)	< 0.0001
Diabetes mellitus (%)	36 (13.9)	54 (26.3)	0.001	20 (12.0)	92 (24.9)	< 0.0001
Hyperlipidemia (%)	66 (25.5)	78 (38.0)	0.004	35 (21.1)	103 (27.9)	0.095
BMI (kg/m^2, mean ±SD)	24.3±3.3	23.9±3.0	0.215[a]	24.2±3.2	24.3±3.2	0.653
HDL-C (mg/dl, mean ±SD)	46.5±16.6	44.7±18.2	0.075	47.0±11.0	47.0±11.0	0.249
Homocysteine (μmol/L, mean ±SD)	9.6±3.5	11.1±4.9	0.001	10.5±4.9	11.2±7.5	0.379
Folate (nmol/L, mean±SD)	9.3±8.7	6.6±5.1	< 0.0001	8.2±6.5	7.2±5.2	0.006
Vitamin B12 (pg/ml, mean ±SD)	762.1±819.3	837.1±942.0	0.415	713.2±310.2	687.0±320.1	0.195
Total cholesterol (mg/dl, mean ±SD)	197.4±40.0	193.6±43.2	0.224	188.8±33.6	190.2±38.5	0.632
Triglyceride (mg/dl, mean ±SD)	152.1±92.6	164.3±120.9	0.647	136.6±82.1	144.6±88.1	0.330
PLT ($10^3/\mu\ell$, mean ±SD)	243.0±59.0	255.1±89.9	0.667	243.0±77.0	246.5±86.6	0.915
PT (sec, mean ±SD)	11.8±0.9	11.9±0.7	0.307	11.7±0.7	11.7±1.1	0.779
aPTT (sec, mean ±SD)	34.2±22.5	31.2±4.8	0.207	32.3±10.9	30.0±4.2	0.139
Fibrinogen (mg/dl, mean ±SD)	362.7±91.8	400.0±119.5	0.021	410.6±130.0	433.0±132.4	0.161
Antithrombin III (%, mean ±SD)	86.5±18.4	95.6±21.9	0.005	98.6±52.0	93.6±16.9	0.788
BUN (mg/dl, mean ±SD)	15.7±5.0	15.8±6.3	0.352	16.0±5.0	16.1±7.9	0.483
Uric Acid (mg/dl, mean ±SD)	4.6±1.4	4.8±1.5	0.401	4.6±1.5	4.6±1.6	0.429

SD, standard deviation; BMI, body mass index; HDL-C, high density lipoprotein cholesterol; PLT, platelet; PT, prothrombin time; aPTT, activated partial thromboplastin time; BUN, blood urea nitrogen.

[*] *P*-values were calculated by Mann-Whitney test for continuous variables and chi-square test for categorical variables.

[a] *P*-values were calculated by two-sided t- test for continuous variables.

* Sample 1 was recruited from 2001 to 2006. ** Sample 2 was recruited from 2007 to 2010.

Supplementary Table S12. Genotype frequency of *PAI-1* seven polymorphisms between ischemic stroke patients and control subjects in samples 1 and 2

Genotype	Sample 1**					Sample 2***				
	Controls (n=259)	Stroke patients (n=205)	AOR (95% CI)*	*P*	*FDR-P*	Controls (n=166)	Stroke patients (n=369)	AOR (95% CI)*	*P*	*FDR-P*
PAI-1 -844G>A										
GG	101 (39.0)	63 (30.7)				61 (36.7)	138 (37.4)			
GA	117 (45.2)	108 (52.7)	1.286 (0.829-1.995)	0.262	0.459	74 (44.6)	184 (49.9)	1.009 (0.659-1.547)	0.966	0.966
AA	41 (15.8)	34 (16.6)	1.734 (0.942-3.193)	0.077	0.462	31 (18.7)	47 (12.7)	0.684 (0.384-1.215)	0.195	0.236
Dominant			1.370 (0.908-2.065)	0.133	0.364			0.898 (0.603-1.337)	0.597	0.758
Recessive			1.341 (0.796-2.261)	0.270	0.405			0.612 (0.362-1.037)	0.068	0.459
PAI-1 -675 4G>5G										
4G4G	116 (44.8)	89 (43.4)				66 (39.8)	129 (35.0)			
4G5G	110 (42.5)	83 (40.5)	0.853 (0.560-1.299)	0.458	0.628	78 (47.0)	169 (45.8)	1.184 (0.776-1.808)	0.433	0.758
5G5G	33 (12.7)	33 (16.1)	1.443 (0.783-2.661)	0.240	0.480	22 (13.3)	71 (19.2)	1.586 (0.875-2.876)	0.129	0.236
Dominant			0.943 (0.638-1.396)	0.770	0.799			1.271 (0.855-1.890)	0.236	0.551
Recessive			1.466 (0.839-2.562)	0.179	0.358			1.476 (0.862-2.525)	0.156	0.628
PAI-1 43G>A										
GG	207 (79.9)	181 (88.3)				141 (84.9)	310 (84.0)			
GA	48 (18.5)	21 (10.2)	0.511 (0.284-0.918)	0.025	0.088	22 (13.3)	57 (15.4)	1.263 (0.724-2.204)	0.412	0.758
AA	4 (1.5)	3 (1.5)	1.200 (0.244-5.898)	0.822	0.822	3 (1.8)	2 (0.5)	0.256 (0.041-1.618)	0.148	0.236
Dominant			0.559 (0.321-0.976)	0.041	0.287			1.131 (0.664-1.927)	0.650	0.758
Recessive			1.353 (0.275-6.660)	0.710	0.710			0.247 (0.039-1.559)	0.137	0.088
PAI-1 9785G>A										
GG	240 (92.7)	184 (89.8)				156 (94.0)	347 (94.0)			
GA	18 (6.9)	20 (9.8)	1.248 (0.616-2.526)	0.538	0.628	10 (6.0)	22 (6.0)	1.026 (0.459-2.291)	0.951	0.966
AA	1 (0.4)	1 (0.5)	N/A	N/A		0 (0.0)	0 (0.0)	N/A	N/A	
Dominant			1.182 (0.590-2.370)	0.637	0.799			1.026 (0.459-2.291)	0.951	0.951
Recessive			N/A	N/A				N/A	N/A	
PAI-1 10692T>C										
TT	98 (37.8)	76 (37.1)				61 (36.7)	109 (29.5)			
TC	128 (49.4)	100 (48.8)	1.009 (0.666-1.530)	0.965	0.965	76 (45.8)	187 (50.7)	1.679 (1.076-2.618)	0.022	0.077
CC	33 (12.7)	29 (14.1)	1.245 (0.661-2.345)	0.498	0.747	29 (17.5)	73 (19.8)	1.457 (0.822-2.582)	0.197	0.236
Dominant			1.053 (0.706-1.571)	0.799	0.799			1.597 (1.060-2.408)	0.025	0.088
Recessive			1.204 (0.680-2.133)	0.524	0.629			1.155 (0.700-1.904)	0.573	0.628
PAI-1 11053T>G										
TT	65 (25.1)	64 (31.2)				43 (25.9)	102 (27.6)			
TG	139 (53.7)	81 (39.5)	0.570 (0.355-0.916)	0.020	0.088	78 (47.0)	186 (50.4)	0.964 (0.608-1.527)	0.875	0.966
GG	55 (21.2)	60 (29.3)	1.082 (0.627-1.864)	0.778	0.822	45 (27.1)	81 (22.0)	0.745 (0.437-1.271)	0.281	0.281
Dominant			0.731 (0.473-1.128)	0.156	0.364			0.888 (0.576-1.371)	0.593	0.758
Recessive			1.579 (1.012-2.465)	0.044	0.264			0.737 (0.473-1.148)	0.177	0.965
PAI-1 12068G>A										
GG	96 (37.1)	90 (43.9)				67 (40.4)	100 (27.1)			
GA	135 (52.1)	82 (40.0)	0.729 (0.477-1.113)	0.143	0.334	75 (45.2)	201 (54.5)	2.158 (1.385-3.361)	0.001	0.007
AA	28 (10.8)	33 (16.1)	1.474 (0.792-2.742)	0.221	0.480	24 (14.5)	68 (18.4)	2.014 (1.111-3.650)	0.021	0.126
Dominant			0.841 (0.565-1.251)	0.392	0.686			2.128 (1.408-3.217)	0.0003	0.002
Recessive			1.625 (0.917-2.877)	0.096	0.288			1.342 (0.790-2.281)	0.277	0.088

AOR, adjusted odds ratio; 95% CI, 95% confidence interval; N/A, not applicable; FDR, false discovery rate.

* Adjusted by age, sex, hypertension, diabetes mellitus, hyperlipidemia, and smoking.

** Sample 1 was recruited from 2001 to 2006. *** Sample 2 was recruited from 2007 to 2010.

Supplementary Table S13. Comparison of genotype frequencies of *PAI-1* seven polymorphisms between ischemic stroke subtype with large-artery disease and control subjects in sample 1 and 2

Genotype	Sample 1**					Sample 2***				
	Controls (n=259)	LAD (n=78)	AOR(95% CI)*	*P*	FDR-*P*	Controls (n=166)	LAD (n=122)	AOR(95% CI)*	*P*	FDR-*P*
PAI-1 -844G>A										
GG	101 (39.0)	29 (37.2)	1.000 (reference)			61 (36.7)	52 (42.6)	1.000 (reference)		
GA	117 (45.2)	36 (46.2)	0.812 (0.443-1.489)	0.500	0.775	74 (44.6)	60 (49.2)	0.932 (0.540-1.608)	0.801	0.801
AA	41 (15.8)	13 (16.7)	1.338 (0.587-3.048)	0.489	0.611	31 (18.7)	10 (8.2)	0.310 (0.127-0.759)	0.010	0.050
Dominant			0.930 (0.532-1.623)	0.797	0.920			0.724 (0.430-1.218)	0.223	0.469
Recessive			1.379 (0.668-2.850)	0.385	0.385			0.317 (0.139-0.724)	0.006	0.030
PAI-1 -675 4G>5G										
4G4G	116 (44.8)	32 (41.0)	1.000 (reference)			66 (39.8)	42 (34.4)	1.000 (reference)		
4G5G	110 (42.5)	32 (41.0)	0.877 (0.484-1.588)	0.664	0.775	78 (47.0)	60 (49.2)	1.373 (0.785-2.402)	0.267	0.623
5G5G	33 (12.7)	14 (17.9)	1.896 (0.823-4.369)	0.133	0.260	22 (13.3)	20 (16.4)	1.249 (0.551-2.827)	0.595	0.728
Dominant			1.028 (0.596-1.774)	0.920	0.920			1.347 (0.795-2.284)	0.268	0.469
Recessive			1.865 (0.890-3.911)	0.099	0.242			1.105 (0.550-2.221)	0.778	0.933
PAI-1 43G>A										
GG	207 (79.9)	69 (88.5)	1.000 (reference)			141 (84.9)	103 (84.4)	1.000 (reference)		
GA	48 (18.5)	8 (10.3)	0.482 (0.201-1.157)	0.102	0.357	22 (13.3)	19 (15.6)	1.275 (0.619-2.624)	0.510	0.745
AA	4 (1.5)	1 (1.3)	N/A	N/A	N/A	3 (1.8)	0 (0.0)	N/A	N/A	N/A
Dominant			0.523 (0.228-1.199)	0.126	0.441			1.099 (0.543-2.222)	0.794	0.823
Recessive			N/A	N/A	N/A			N/A	N/A	N/A
PAI-1 9785G>A										
GG	240 (92.7)	71 (91.0)	1.000 (reference)			156 (94.0)	116 (95.1)	1.000 (reference)		
GA	18 (6.9)	7 (9.0)	1.338 (0.505-3.546)	0.558	0.775	10 (6.0)	6 (4.9)	0.780 (0.262-2.328)	0.657	0.767
AA	1 (0.4)	0 (0.0)	N/A	N/A	N/A	0 (0.0)	0 (0.0)	N/A	N/A	N/A
Dominant			1.255 (0.478-3.291)	0.645	0.903			0.780 (0.262-2.328)	0.657	0.823
Recessive			N/A	N/A	N/A			N/A	N/A	N/A
PAI-1 10692T>C										
TT	98 (37.8)	27 (34.6)	1.000 (reference)			61 (36.7)	36 (29.5)	1.000 (reference)		
TC	128 (49.4)	37 (47.4)	1.025 (0.568-1.852)	0.934	0.934	76 (45.8)	68 (55.7)	2.106 (1.166-3.803)	0.014	0.049
CC	33 (12.7)	14 (17.9)	1.831 (0.794-4.222)	0.156	0.260	29 (17.5)	18 (14.8)	1.161 (0.501-2.687)	0.728	0.728
Dominant			1.163 (0.664-2.036)	0.598	0.903			1.767 (1.014-3.077)	0.044	0.154
Recessive			1.760 (0.844-3.671)	0.132	0.242			0.705 (0.353-1.410)	0.323	0.538
PAI-1 11053T>G										
TT	65 (25.1)	27 (34.6)	1.000 (reference)			43 (25.9)	31 (25.4)	1.000 (reference)		
TG	139 (53.7)	29 (37.2)	0.470 (0.246-0.898)	0.022	0.154	78 (47.0)	63 (51.6)	1.213 (0.663-2.220)	0.532	0.745
GG	55 (21.2)	22 (28.2)	0.853 (0.404-1.802)	0.677	0.677	45 (27.1)	28 (23.0)	0.740 (0.351-1.563)	0.430	0.717
Dominant			0.587 (0.326-1.056)	0.075	0.441			1.069 (0.599-1.907)	0.823	0.823
Recessive			1.389 (0.755-2.553)	0.291	0.364			0.657 (0.361-1.198)	0.171	0.428
PAI-1 12068G>A										
GG	96 (37.1)	26 (33.3)	1.000 (reference)			67 (40.4)	35 (28.7)	1.000 (reference)		
GA	135 (52.1)	38 (48.7)	1.282 (0.697-2.358)	0.424	0.775	75 (45.2)	69 (56.6)	2.663 (1.450-4.891)	0.002	0.014
AA	28 (10.8)	14 (17.9)	2.234 (0.937-5.322)	0.070	0.260	24 (14.5)	18 (14.8)	1.764 (0.756-4.119)	0.190	0.475
Dominant			1.416 (0.798-2.514)	0.235	0.548			2.335 (1.327-4.111)	0.003	0.021
Recessive			1.743 (0.825-3.684)	0.145	0.242			0.970 (0.474-1.986)	0.933	0.933

AOR, adjusted odds ratio; 95% CI, 95% confidence interval; LAD, large-artery disease; N/A, not applicable; FDR, false discovery rate.

* Adjusted by age, sex, hypertension, diabetes mellitus, hyperlipidemia, and smoking.

** Sample 1 was recruited from 2001 to 2006. *** Sample 2 was recruited from 2007 to 2010.

Supplementary Table S14. Comparison of genotype frequencies of *PAI-1* seven polymorphisms between ischemic stroke subtype with small-vessel disease and control subjects in sample 1 and 2

Genotype	Sample 1**					Sample 2***				
	Controls (n=259)	SVD (n=42)	AOR(95% CI)*	*P*	FDR-*P*	Controls (n=166)	SVD (n=98	AOR(95% CI)*	*P*	FDR-*P*
PAI-1 -844G>A										
GG	101 (39.0)	14 (33.3)	1.000 (reference)			61 (36.7)	32 (32.7)	1.000 (reference)		
GA	117 (45.2)	22 (52.4)	0.865 (0.376-1.986)	0.731	0.867	74 (44.6)	49 (50.0)	1.291 (0.716-2.330)	0.396	0.774
AA	41 (15.8)	6 (14.3)	1.722 (0.514-5.764)	0.378	0.854	31 (18.7)	17 (17.3)	1.085 (0.504-2.335)	0.835	0.835
Dominant			0.983 (0.458-2.112)	0.965	0.974			1.177 (0.680-2.036)	0.561	0.785
Recessive			1.431 (0.529-3.872)	0.480	0.887			0.873 (0.437-1.744)	0.701	0.701
PAI-1 -675 4G>5G										
4G4G	116 (44.8)	18 (42.9)	1.000 (reference)			66 (39.8)	37 (37.8)	1.000 (reference)		
4G5G	110 (42.5)	20 (47.6)	0.918 (0.417-2.021)	0.831	0.867	78 (47.0)	39 (39.8)	0.838 (0.468-1.501)	0.553	0.774
5G5G	33 (12.7)	4 (9.5)	1.321 (0.347-5.019)	0.683	0.854	22 (13.3)	22 (22.4)	1.566 (0.734-3.343)	0.246	0.318
Dominant			0.905 (0.428-1.915)	0.794	0.974			0.993 (0.580-1.699)	0.979	0.979
Recessive			1.091 (0.328-3.627)	0.887	0.887			1.686 (0.857-3.317)	0.131	0.328
PAI-1 43G>A										
GG	207 (79.9)	40 (95.2)	1.000 (reference)			141 (84.9)	86 (87.8)	1.000 (reference)		
GA	48 (18.5)	2 (4.8)	0.265 (0.058-1.209)	0.086	0.602	22 (13.3)	12 (12.2)	0.867 (0.395-1.905)	0.722	0.842
AA	4 (1.5)	0 (0.0)	N/A	N/A	N/A	3 (1.8)	0 (0.0)	N/A	N/A	N/A
Dominant			0.258 (0.057-1.172)	0.079	0.553			0.746 (0.345-1.615)	0.457	0.785
Recessive			N/A	N/A	N/A			N/A	N/A	N/A
PAI-1 9785G>A										
GG	240 (92.7)	38 (90.5)	1.000 (reference)			156 (94.0)	92 (93.9)	1.000 (reference)		
GA	18 (6.9)	4 (9.5)	1.113 (0.316-3.915)	0.867	0.867	10 (6.0)	6 (6.1)	0.944 (0.321-2.776)	0.916	0.916
AA	1 (0.4)	0 (0.0)	N/A	N/A	N/A	0 (0.0)	0 (0.0)	N/A	N/A	
Dominant			1.086 (0.311-3.795)	0.897	0.974			0.944 (0.321-2.776)	0.916	0.979
Recessive			N/A	N/A	N/A			N/A	N/A	
PAI-1 10692T>C										
TT	98 (37.8)	16 (38.1)	1.000 (reference)			61 (36.7)	23 (23.5)	1.000 (reference)		
TC	128 (49.4)	23 (54.8)	1.211 (0.567-2.585)	0.621	0.867	76 (45.8)	48 (49.0)	1.730 (0.917-3.265)	0.091	0.319
CC	33 (12.7)	3 (7.1)	1.007 (0.225-4.516)	0.992	0.992	29 (17.5)	27 (27.6)	2.203 (1.048-4.629)	0.037	0.185
Dominant			1.160 (0.548-2.453)	0.699	0.974			1.854 (1.030-3.336)	0.040	0.140
Recessive			0.718 (0.190-2.705)	0.624	0.887			1.660 (0.891-3.091)	0.111	0.328
PAI-1 11053T>G										
TT	65 (25.1)	13 (31.0)	1.000 (reference)			43 (25.9)	32 (32.7)	1.000 (reference)		
TG	139 (53.7)	19 (45.2)	0.649 (0.273-1.543)	0.328	0.867	78 (47.0)	47 (48.0)	0.808 (0.439-1.486)	0.492	0.774
GG	55 (21.2)	10 (23.8)	0.719 (0.247-2.095)	0.545	0.854	45 (27.1)	19 (19.4)	0.653 (0.314-1.357)	0.254	0.318
Dominant			0.670 (0.300-1.496)	0.328	0.974			0.758 (0.430-1.337)	0.339	0.785
Recessive			1.127 (0.474-2.677)	0.787	0.887			0.748 (0.400-1.396)	0.361	0.565
PAI-1 12068G>A										
GG	96 (37.1)	18 (42.9)	1.000 (reference)			67 (40.4)	26 (26.5)	1.000 (reference)		
GA	135 (52.1)	19 (45.2)	0.913 (0.415-2.007)	0.821	0.867	75 (45.2)	53 (54.1)	1.914 (1.043-3.515)	0.036	0.252
AA	28 (10.8)	5 (11.9)	1.462 (0.416-5.137)	0.554	0.854	24 (14.5)	19 (19.4)	1.976 (0.904-4.317)	0.088	0.220
Dominant			1.013 (0.480-2.135)	0.974	0.974			1.940 (1.099-3.426)	0.022	0.140
Recessive			1.419 (0.458-4.397)	0.544	0.887			1.303 (0.654-2.597)	0.452	0.565

AOR, adjusted odds ratio; 95% CI, 95% confidence interval; SVD, small-vessel disease; N/A, not applicable; FDR, false discovery rate.

* Adjusted by age, sex, hypertension, diabetes mellitus, hyperlipidemia, and smoking.

** Sample 1 was recruited from 2001 to 2006. *** Sample 2 was recruited from 2007 to 2010.

Supplementary Table S15. Comparison of genotype frequencies of *PAI-1* seven polymorphisms between ischemic stroke subtype with cardioembolism and control subjects in sample 1 and 2

Genotype	Sample 1**					Sample 2***				
	Controls (n=259)	CE (n=21)	AOR(95% CI)*	*P*	FDR-*P*	Controls (n=166)	CE (n=31)	AOR(95% CI)*	*P*	FDR-*P*
PAI-1 -844G>A										
GG	101 (39.0)	6 (28.6)	1.000 (reference)			61 (36.7)	14 (45.2)	1.000 (reference)		
GA	117 (45.2)	13 (61.9)	1.504 (0.519-4.355)	0.452	0.981	74 (44.6)	11 (35.5)	0.377 (0.140-1.016)	0.054	0.324
AA	41 (15.8)	2 (9.5)	0.869 (0.155-4.856)	0.873	0.873	31 (18.7)	6 (19.4)	0.910 (0.289-2.868)	0.871	0.871
Dominant			1.393 (0.501-3.874)	0.525	0.859			0.505 (0.215-1.186)	0.117	0.702
Recessive			0.613 (0.135-2.788)	0.527	0.659			1.266 (0.441-3.635)	0.662	0.763
PAI-1 -675 4G>5G										
4G4G	116 (44.8)	7 (33.3)	1.000 (reference)			66 (39.8)	10 (32.3)	1.000 (reference)		
4G5G	110 (42.5)	8 (38.1)	1.013 (0.344-2.990)	0.981	0.981	78 (47.0)	13 (41.9)	1.131 (0.444-2.886)	0.796	0.955
5G5G	33 (12.7)	6 (28.6)	3.788 (1.059-13.545)	0.041	0.205	22 (13.3)	8 (25.8)	2.029 (0.658-6.260)	0.218	0.812
Dominant			1.522 (0.585-3.960)	0.390	0.859			1.372 (0.587-3.206)	0.465	0.904
Recessive			3.883 (1.294-11.654)	0.016	0.080			2.003 (0.746-5.377)	0.168	0.740
PAI-1 43G>A										
GG	207 (79.9)	17 (81.0)	1.000 (reference)			141 (84.9)	25 (80.6)	1.000 (reference)		
GA	48 (18.5)	4 (19.0)	1.105 (0.349-3.500)	0.865	0.981	22 (13.3)	6 (19.4)	1.737 (0.600-5.024)	0.308	0.924
AA	4 (1.5)	0 (0.0)	N/A	N/A	N/A	3 (1.8)	0 (0.0)	N/A	N/A	N/A
Dominant			1.030 (0.326-3.253)	0.960	0.960			1.514 (0.532-4.313)	0.437	0.904
Recessive			N/A	N/A	N/A			N/A	N/A	N/A
PAI-1 9785G>A										
GG	240 (92.7)	20 (95.2)	1.000 (reference)			156 (94.0)	31 (100.0)	1.000 (reference)		
GA	18 (6.9)	1 (4.8)	0.728 (0.090-5.892)	0.766	0.981	10 (6.0)	0 (0.0)	N/A	N/A	N/A
AA	1 (0.4)	0 (0.0)	N/A	N/A	N/A	0 (0.0)	0 (0.0)	N/A	N/A	N/A
Dominant			0.698 (0.087-5.625)	0.736	0.859			N/A	N/A	N/A
Recessive			N/A	N/A	N/A			N/A	N/A	N/A
PAI-1 10692T>C										
TT	98 (37.8)	7 (33.3)	1.000 (reference)			61 (36.7)	10 (32.3)	1.000 (reference)		
TC	128 (49.4)	10 (47.6)	1.097 (0.400-3.006)	0.858	0.981	76 (45.8)	18 (58.1)	1.164 (0.466-2.904)	0.745	0.955
CC	33 (12.7)	4 (19.0)	2.394 (0.578-9.910)	0.228	0.380	29 (17.5)	3 (9.7)	0.495 (0.113-2.168)	0.351	0.812
Dominant			1.251 (0.479-3.269)	0.648	0.859			1.028 (0.434-2.435)	0.951	0.951
Recessive			1.809 (0.550-5.946)	0.329	0.548			0.494 (0.132-1.855)	0.296	0.740
PAI-1 11053T>G										
TT	65 (25.1)	7 (33.3)	1.000 (reference)			43 (25.9)	7 (22.6)	1.000 (reference)		
TG	139 (53.7)	11 (52.4)	0.597 (0.208-1.718)	0.339	0.981	78 (47.0)	15 (48.4)	0.974 (0.344-2.759)	0.960	0.960
GG	55 (21.2)	3 (14.3)	0.613 (0.160-2.351)	0.476	0.595	45 (27.1)	9 (29.0)	1.503 (0.477-4.737)	0.487	0.812
Dominant			0.638 (0.241-1.688)	0.365	0.859			1.168 (0.444-3.073)	0.753	0.904
Recessive			0.857 (0.274-2.677)	0.790	0.790			1.299 (0.530-3.186)	0.567	0.763
PAI-1 12068G>A										
GG	96 (37.1)	7 (33.3)	1.000 (reference)			67 (40.4)	11 (35.5)	1.000 (reference)		
GA	135 (52.1)	10 (47.6)	1.089 (0.395-3.002)	0.869	0.981	75 (45.2)	15 (48.4)	1.198 (0.478-3.003)	0.701	0.955
AA	28 (10.8)	4 (19.0)	2.357 (0.592-9.387)	0.224	0.380	24 (14.5)	5 (16.1)	1.247 (0.364-4.276)	0.726	0.871
Dominant			1.274 (0.489-3.317)	0.621	0.859			1.202 (0.519-2.779)	0.668	0.904
Recessive			1.901 (0.576-6.274)	0.292	0.548			1.189 (0.386-3.664)	0.763	0.763

AOR, adjusted odds ratio; 95% CI, 95% confidence interval; CE, cardioembolism; N/A, not applicable; FDR, false discovery rate.

* Adjusted by age, sex, hypertension, diabetes mellitus, hyperlipidemia, and smoking.

** Sample 1 was recruited from 2001 to 2006. *** Sample 2 was recruited from 2007 to 2010.

Supplementary Table S16. Clinical variables in ischemic stroke patients stratified by *PAI-1* -844GA/-675 5G genotype

Genotype	Homocysteine (mmol/L)		Folate (mg/ml)		Vitamin B12 (pg/ml)		Total cholesterol (mg/dl)		Triglyceride (mg/dl)		PLT ($10^3/\mu\ell$)		BMI (kg/m^2)	
	Mean ± SD	P^a	Mean ± SD	P^a	Mean ± SD	P^a	Mean ± SD	P^a	Mean ± SD	P^a	Mean ± SD	P^a	Mean ± SD	P^a
PAI-1-844 G>A/-675 4G>5G(N)														
GG+4G4G(79)	10.02±4.10	0.976	7.48±4.50	0.420	716.39±378.36	0.248	189.04±36.12	**0.013**	143.67±105.36	0.143	253.37±129.99	0.489	23.23±3.25	**0.011**
GA+5G5G(20)	9.99±2.97		6.55±4.06		910.37±1227.04		212.53±35.81		184.11±111.67		232.75±47.03		25.66±4.03	

Genotype	PT (sec)		aPTT (sec)		Fibrinogen (mg/dl)		Antithrombin III (%)		BUN (mg/dl)		Uric acid (mg/dl)		HDL-cholesterol ($10^3/\mu\ell$)	
	Mean ± SD	P^a	Mean ± SD	P^a	Mean ± SD	P^a	Mean ± SD	P^a	Mean ± SD	P^a	Mean ± SD	P^a	Mean ± SD	P^a
PAI-1-844 G>A/-675 4G>5G														
GG+4G4G(79)	11.92±1.06	0.236	32.37±10.34	0.376	424.68±149.96	0.628	91.77±18.51	0.418	16.38±5.99	0.168	4.35±1.39	**0.041**	44.40±17.70	0.829
GA+5G5G(20)	11.62±0.66		30.25±4.53		404.31±132.60		96.28±23.50		14.45±3.13		5.12±1.73		45.44±13.64	

PLT, platelet; BMI, body mass index; PT, prothrombin time; aPTT, activated partial thromboplastin time; HDL-cholesterol, high-density lipoprotein cholesterol; SD, standard deviation. [a] Calculated using Student's t-test

.
